# Grass lignin: biosynthesis, biological roles, and industrial applications

**DOI:** 10.3389/fpls.2024.1343097

**Published:** 2024-02-23

**Authors:** Luigi M. Peracchi, Rahele Panahabadi, Jaime Barros-Rios, Laura E. Bartley, Karen A. Sanguinet

**Affiliations:** ^1^ Department of Crop and Soil Sciences, Washington State University, Pullman, WA, United States; ^2^ Institute of Biological Chemistry, Washington State University, Pullman, WA, United States; ^3^ Division of Plant Sciences and Interdisciplinary Plant Group, University of Missouri, Columbia, MO, United States

**Keywords:** lignin, hydroxycinnamic acids, grass, cereals, abiotic stress, biotic stress, monolignol transport, monolignol polymerization

## Abstract

Lignin is a phenolic heteropolymer found in most terrestrial plants that contributes an essential role in plant growth, abiotic stress tolerance, and biotic stress resistance. Recent research in grass lignin biosynthesis has found differences compared to dicots such as *Arabidopsis thaliana*. For example, the prolific incorporation of hydroxycinnamic acids into grass secondary cell walls improve the structural integrity of vascular and structural elements via covalent crosslinking. Conversely, fundamental monolignol chemistry conserves the mechanisms of monolignol translocation and polymerization across the plant phylum. Emerging evidence suggests grass lignin compositions contribute to abiotic stress tolerance, and periods of biotic stress often alter cereal lignin compositions to hinder pathogenesis. This same recalcitrance also inhibits industrial valorization of plant biomass, making lignin alterations and reductions a prolific field of research. This review presents an update of grass lignin biosynthesis, translocation, and polymerization, highlights how lignified grass cell walls contribute to plant development and stress responses, and briefly addresses genetic engineering strategies that may benefit industrial applications.

## 1 Introduction

Given the global dietary and economic importance of cereal grains as well as the UN projected population increase to 9.7 billion people by 2050 (UN, 2022), research is underway to increase cereal yields while maintaining ecosystem services (Rockstrom et al., 2020). Likewise, increasing populations increase energy demands, which drives ongoing research to develop environmentally and economically sustainable biofuels and biochemicals (Chu and Majumdar, 2012). Research into improving cereal yields and sustainable biofuels may converge at the composition of grass cell walls. Research in cereals seeks to improve stress tolerance and sustain yields, which in some cases improves grass cell wall integrity, while much biofuel research seeks to minimize grass cell wall recalcitrance to maximize release of sugars from feedstocks (i.e., saccharification). Both goals hinge on either the improvement or reduction of the second most prolific cell wall polymer on earth: lignin.

Lignin, a primary component of feedstock recalcitrance to saccharification (Zeng et al., 2014), also provides critical structural reinforcement and hydrophobicity to plant tracheary elements (Fan et al., 2006; Chmielewska et al., 2016), rigidity to structural fibers to resist gravity (Li et al., 2022a), and resistance to enzymatic and mechanical pathogen invasion (Nicholson and Hammerschmidt, 1992; Bellincampi et al., 2014; Lee et al., 2019). Grass lignin is a complex hydrophobic hetero-phenolic polymer primarily composed of three phenolic alcohols: *p*-coumaryl alcohol (H), coniferyl alcohol (G), and sinapyl alcohol (S); two hydroxycinnamic acids: *p*-coumaric acid (*p*CA) and ferulic acid (FA); four hydroxycinnamate-phenolic conjugates: γ-*p*-coumaroylated coniferyl alcohol (*p*CA-G) and γ-*p*-coumaroylated sinapyl alcohol (*p*CA-S), feruloylated coniferyl alcohol (G-FA), and feruloyl sinapyl alcohol (S-FA); and thus far one known flavone: tricin, and many additional conjugated derivatives found in lower concentrations (Ralph, 2010; del Rio et al., 2012; Lan et al., 2015; Karlen et al., 2016; del Río et al., 2022). Grass lignin monomers are synthesized in the phenylpropanoid and flavone pathways, pass through the cell membrane, and are polymerized in secondary cell walls (Ralph et al., 2019; Tobimatsu and Schuetz, 2019). The purpose of this review is threefold: to present the current models of grass lignin synthesis, translocation, and polymerization; to highlight how lignin contributes to grass stress resistance; and to suggest future avenues of research that may benefit both cereal breeders and valorization engineers.

## 2 Grass lignin biosynthesis

### 2.1 Phenylpropanoid and flavone enzymes

#### 2.1.1 Substrate promiscuity and subfunctionalization in grass monolignol biosynthesis

**Figure 1:**
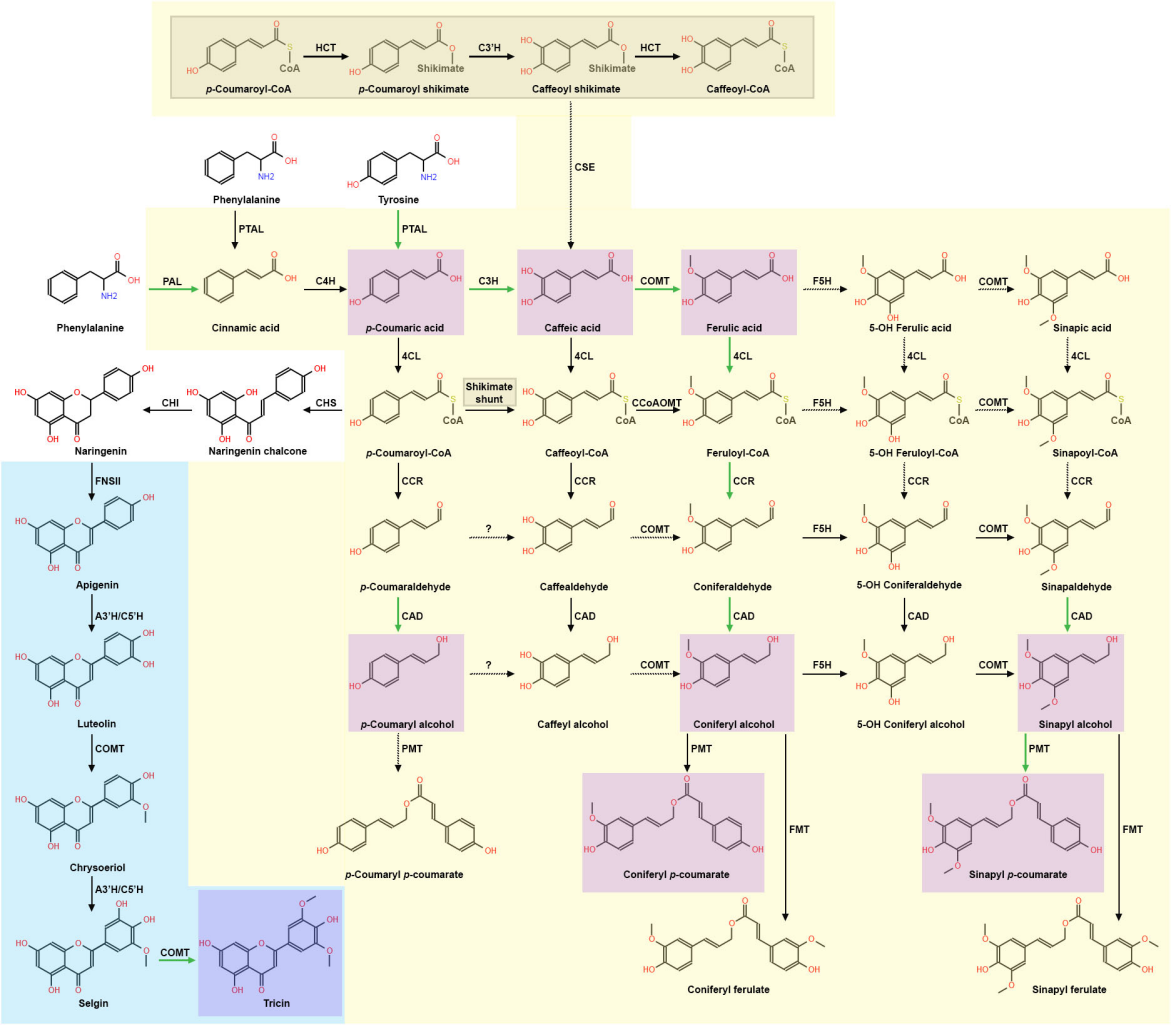
The current model of grass lignin biosynthesis, which includes aspects of the phenylpropanoid pathway and the flavone pathway. Grass preferential carbon flux is shown with green arrows, while dashed arrows represent unverified steps in the pathways. The current grass phenylpropanoid synthesis model is presented with a yellow background. The “shikimate shunt” is expanded on in the grey shaded region and is part of the phenylpropanoid model. The current monocot flavone synthesis model leading to formation of tricin is presented with a blue background. Monocot monolignols and conjugates found in significant concentrations are highlighted in purple. Phenylpropanoid enzymes: PAL, phenylalanine ammonia lyase; PTAL, phenylalanine/tyrosine ammonia lyase; C4H, cinnamate 4-hydroxylase; 4CL, 4-hydroxycinnamate:CoA ligase; C3H, 4-coumarate 3-hydroxylase/ascorbate peroxidase; CSE, caffeoyl shikimate esterase; HCT, hydroxycinnamoyl CoA: shikimate/quinate hydroxycinnamoyl transferase; C3´H, 4-coumaroyl shikimate 3´-hydroxylase; CCoAOMT, caffeoyl CoA 3-O-methyltransferase; CCR, cinnamoyl CoA reductase; F5H, ferulate/coniferaldehyde 5-hydroxylase; COMT, caffeate/5-hydroxyferulate 3-O-methyltransferase; CAD, cinnamyl alcohol dehydrogenase; PMT, *p*-coumaroyl-CoA: monolignol transferase; FMT, feruloyl-CoA: monolignol transferase. Flavonoid and flavone-related enzymes: CHS, chalcone synthase; CHI, chalcone isomerase; FNSII, flavone synthase II; A3’H/C5’H, apigenin 3′-hydroxylase/chrysoeriol 5′-hydroxylase.

Lignin monomers are synthesized in the phenylpropanoid pathway, general models of which have been extensively reviewed (Vanholme et al., 2019; Barros et al., 2019a). However, grass phenylpropanoid biosynthesis research is revealing unique features that diverge from the dicot model, thus leading to a monolignol synthesis model for grasses (**Figure 1**). For instance, the dicot model suggests phenylalanine ammonia lyase (PAL), which deaminates shikimate-derived phenylalanine into cinnamic acid, to be the singular entry point and rate-limiting step in phenylpropanoid biosynthesis (Barros and Dixon, 2020; Yao et al., 2021). In *Brachypodium distachyon, Sorghum bicolor, Triticum aestivum, Bambusa oldhamii*, and *Zea mays*, a bifunctional PAL homolog named phenylalanine-tyrosine ammonia-lyase (PTAL) deaminates both phenylalanine and tyrosine in parallel with PAL (Rosler et al., 1997; Barros et al., 2016; Godin et al., 2016; Jun et al., 2018; Feduraev et al., 2020; Hsieh et al., 2021; Simpson et al., 2021). In addition, PTAL preferentially deaminates tyrosine into *p*CA, effectively bypassing 4-hydroxylase (C4H) conversion of PAL-derived cinnamic acid to *p*CA and generating almost half of the total lignin detected (Barros et al., 2016). *p*CA pools can be converted to *p*-coumaroyl-CoA via 4-hydroxycinnamate:CoA ligase (4CL) or caffeic acid (CAF) via coumarate 3-hydroxylase (C3H), which has recently been observed in *B. distachyon, A. thaliana*, and *S. bicolor* to be an ascorbate peroxidase (Barros et al., 2019a; Zhang et al., 2023b).

Interestingly, recent evidence suggests grasses may preferentially shunt phenylpropanoid metabolism to hydroxycinnamic acid synthesis (**Figure 1**). Although grasses use both 4CL and C3H metabolic pathways, high concentrations of PTAL, C3H, and COMT were found in the same fraction derived from *B. distachyon* stem tissue, which led the authors to postulate that PTAL, C3H, and COMT may form a protein complex that contributes to the observed preferential metabolic flux into hydroxycinnamic acid synthesis (Barros et al., 2019a). ^13^C flux analysis from the same study suggests *B. distachyon* caffeate 3-*O*-methyltransferase (COMT) preferentially methylates CAF, generating significantly more FA compared to the *A. thaliana* COMT ortholog. Furthermore, analysis of metabolic profiles of mutants in the shikimate intermediate pathway enzymes, hydroxycinnamoyl CoA: shikimate/quinate hydroxycinnamoyl transferase (HCT) and 4-coumaroyl shikimate 3′-hydroxylase (C3’H), resulted in reduced lignin content, but wildtype growth (Barros et al., 2022). In contrast, disruption of *A. thaliana hct* and *c3’h* resulted in significantly reduced total lignin and severe dwarfism (Franke et al., 2002a, b; Hoffmann et al., 2004). These data and the previous observation of preferential phenylpropanoid flux into hydroxycinnamic acid metabolism in *B. distachyon* (Barros et al., 2019a) led to the hypothesis that the phenylpropanoid shikimate intermediate pathway is not essential for *B. distachyon* development (Barros et al., 2022). In support of this hypothesis, caffeoyl shikimate esterase (CSE) activity is crucial for *A. thaliana*, *Medicago truncatula*, and hybrid poplar (*Populus tremula* × *Populus alba*) development (Vanholme et al., 2013; Saleme et al., 2017). However, phylogenetic analyses have not identified clear CSE homologs in *B. distachyon, O. sativa, Z. mays*, *Setaria italica*, *S. bicolor*, *Oryza minuta*, *Aegilops tauschii*, or *Hordeum vulgare* (Ha et al., 2016; Serrani-Yarce et al., 2021). A surprising exception is *Panicum virgatum*, in which both phylogenetic evidence and enzymatic activity of CSE has been detected, leaving the metabolic purpose of CSE in monocots unclear (Ha et al., 2016; Serrani-Yarce et al., 2021). Continued investigation of the functional significance of the shikimate pathway in more grass species will further test the hypothesis of the shikimate pathway being an alternative phenylpropanoid flux route that may provide protective metabolic redundancy (Barros et al., 2022).

Following the action of 4CL, CoA groups are removed from the γ carbon by cinnamoyl-CoA reductase (CCR), resulting in production of cinnamaldehydes. Upstream phenylpropanoid-CoA concentrations likely drive preferential flux through feruloyl-CoA metabolism (Barros et al., 2022) (**Figure 1**). Multiple CCR isoforms showing differing substrate specificities are found co-expressed in *P. virgatum* (Escamilla-Trevino et al., 2010), *O. sativa* (Park et al., 2017), and *S. bicolor* (Sattler et al., 2017), thus likely providing an additional measure of protective metabolic redundancy. This protective metabolic redundancy was inadvertently demonstrated via single gene disruption of feruloyl-CoA-specific *ZmCCR1* in *Z. mays* (Smith et al., 2017a). Despite the *Zmccr1* mutant showing a reported 40% decrease in H monomers, 56% decrease in G monomers, and a 26% decrease in S monomers with WT (1.7:40:59) versus mutant (1.6:27:71) H:G:S ratios, the *Zmccr1* mutant showed no significant growth defects. These data support that feruloyl-CoA metabolism is preferential in grasses due to upstream phenylpropanoid pools, and that co-expressed CCR isoforms can compensate for *CCR* homolog disruption.

Downstream in the phenylpropanoid biosynthesis pathway, S monomers are sequentially generated by the phenylpropanoid enzyme ferulate 5-hydroxylase F5H [also known as coniferaldehyde-5-hydroxylase (CAld5H)] and COMT (Takeda et al., 2017, 2019; Wu et al., 2019). In grasses, F5H primarily converts coniferaldehyde to 5-hydroxyconiferaldehyde, and was observed to be the rate-limiting step of S monomer synthesis in *P. virgatum* (Wu et al., 2019). However, a *f5h* null mutant generated in a *O. sativa* RNAi-*comt* background unexpectedly produced significant amounts of *p*CA-S monomers, suggesting that an unidentified enzyme or uncharacterized F5H isoform may generate a separate and discrete pool of 5-hydroxyconiferaldehyde (Takeda et al., 2019). These data led to the hypothesis that a grass-specific lignin pathway may exist to produce *p*CA-S monomers independently of the current phenylpropanoid biosynthesis model (Takeda et al., 2019; Barros and Dixon, 2020; Shafiei et al., 2023). Lack of *F5H*-related functional studies in grasses raises new and exciting questions, such as: is the observed separate pool of *p*CA-S monomers unique to *O. sativa* or common in all grasses; and is there a biological function of an alternate S monomer biosynthetic pathway?

The final step of the lignin biosynthetic pathway is performed by cinnamyl alcohol dehydrogenase (CAD), which reduces cinnamaldehydes into cinnamyl alcohols: *p*-coumaraldehyde is reduced to H monomers, coniferaldehyde is reduced to G monomers, and sinapaldehyde is reduced to S monomers (Vanholme et al., 2010; del Río et al., 2022). Predominant CAD isoforms in C4 grasses *S. bicolor* (Jun et al., 2017) and *P. virgatum* (Saathoff et al., 2011b, 2012) have reportedly greater substrate specificity for sinapaldehyde; whereas, primary CAD isoforms in C3 grasses *T. aestivum* (Ma, 2010) and *O. sativa* (Park et al., 2018) have reportedly greater substrate specificity for coniferaldehyde. Genetic mutations of critical phenylpropanoid genes such as *CAD* in C4 grasses are easily identifiable due to a conspicuous brown midrib phenotype, observed in *S. bicolor* (Saballos et al., 2009; Sattler et al., 2009; Li and Huang, 2017), *Z. mays* (Halpin et al., 1998; Chen et al., 2012b; Liu et al., 2021b), and *P. virgatum* (Saathoff et al., 2011a). To date, characterized *cad* mutants show similar phenotypes such as decreased total lignin and increased saccharification efficiency (Zeng et al., 2014; Carmona et al., 2015; Zhong et al., 2021; del Río et al., 2022). However, *cad1* mutations in C3 grasses such as *B. distachyon*, *O. sativa*, *H. vulgare*, and *T. aestivum* do not present a brown midrib, but display increased reddish coloration in various tissues such as the nodes, spike, rachilla, and lemma (d’Yvoire et al., 2013). It is still unclear why the brown midrib phenotype appears in C4 grasses, but not in C3 grasses (Sattler et al., 2010; Eloy et al., 2017). A recent phenolic analysis of *zmcad2* in maize showed a significant increase in integration of cinnamaldehydes into lignin and of soluble compounds conjugated with FA, SA, vanillic acid, and hydroxybenzoyl hexose in the mutant (Liu et al., 2021b). These data led to the hypothesis of an uncharacterized metabolic sink that may act as an upper limit on production of FA monomer synthesis, which in turn may moderate diFA-mediated cell wall crosslinking.

#### 2.1.2 Hydroxycinnamic acids and their conjugates

In grasses, both xylan and lignin polymers may harbor substitutions with hydroxycinnamic acids such as ferulic acid (FA) or *p*-coumaric acid (*p*CA). Hydroxycinnamic groups are either esterified or etherified to lignin (Hatfield et al., 2017). Feruloylation of xylan facilitates covalent cross linkages of xylan chains or xylan–lignin via diferulate (diFA) bridge formation (Hatfield et al., 2017; Terrett and Dupree, 2019; Feijao et al., 2022). In a recent study, biochemical and histochemical evidence from *B. distachyon* culms suggest that structural variation in xylan molecules may determine interactions with both lignin and other cell wall components to confer functional specialization (Tryfona et al., 2023).

Recent metabolomic and genetic evidence suggests phenylpropanoid synthesis is preferentially shunted towards hydroxycinnamic acid synthesis in grasses (Ralph, 2010; Karlen et al., 2018; Barros et al., 2022). Thus, it is not surprising that greater amounts of *p*CA and FA are found in grass cell walls compared to dicots (**Figure 1**). *p*CA is a precursor metabolite for most phenylpropanoids and flavonoids (Vogt, 2010). *p*CA is also abundantly conjugated to S and G monomers via *p*-coumaroyl-CoA: monolignol acyltransferases (PMT), which are part of the larger “BAHD” acyltransferase protein family (Grabber et al., 1996; Withers et al., 2012; Marita et al., 2014; Petrik et al., 2014; Chandrakanth et al., 2023). The function of *p*CA-monolignol conjugates is currently unknown (Hatfield et al., 2017), but two hypothesis exist: 1) *p*CA acts to transfer radicals during S monomer polymerization (Ralph et al., 2004a), and 2) *p*CA-S monomers terminate polymerization, leading to a more linearized lignin polymer and subsequently change lignin polymer properties (Kishimoto et al., 2005; Hatfield et al., 2017). In addition to conjugating to S monomers, *p*CA is also acylated to arabinoxylans (AXs) in grass cell walls via BAHD acyltransferases in *O. sativa* (Bartley et al., 2013), *S. viridis* (Mota et al., 2021), and *P. virgatum* (Li et al., 2018a).

FA is also found in grass cell walls at high concentrations relative to dicots (Ralph, 2010). FA is predominantly found esterified to AX sidechains in grass cell walls, but has also been found ether-linked to monolignols in low concentrations (de Oliveira et al., 2015). Recent genetic and metabolomic evidence suggest BAHD acyltransferases may significantly contribute to grass AX feruloylation (de Souza et al., 2018, 2019). Observed AX feruloylation during early stages of secondary cell wall synthesis has led to the hypothesis that FA monomers ester-bound to AX act as nucleation sites for lignin polymerization (Ralph et al., 1995; Grabber et al., 2004; Ralph, 2010; Zhang et al., 2019). DiFA is formed via oxidative radical coupling of FA monomers, facilitated by both laccases and peroxidases (Ralph et al., 1994; Quideau and Ralph, 1997; Carunchio et al., 2001; Ward et al., 2001). DiFAs often link local AX polymers together, resulting in the lignocellulose crosslinking that is characteristic of grass cell walls (Ralph et al., 1995; Grabber et al., 2000; Ralph, 2010; Eugene et al., 2020; del Río et al., 2022). DiFA-mediated cell wall crosslinking has been strongly correlated with greater structural integrity of the cell wall (Jung and Phillips, 2010; Hatfield et al., 2018) and may contribute to both fungal pathogen (Piotrowski et al., 2015) and antinutritional herbivory defense (Barros-Rios et al., 2015). Lastly, recent findings suggest BAHD enzymes with feruloyl-CoA monolignol transferase (FMT) activity may also contribute to FA-monolignol conjugation (Wilkerson et al., 2014; Karlen et al., 2016; Smith et al., 2022).

#### 2.1.3 Promiscuous flavone grass enzymes synthesize the lignin monomer tricin

Tricin was recently determined to be the first non-phenylpropanoid lignin monomer found in both commelinid and non-commelinid monocots (del Rio et al., 2012; Lan et al., 2015; Lan et al., 2016a, b). Tricin has also been detected in dicot legumes *Medicago sativa* and *M. truncatula*; synthesis of which is currently hypothesized to result from convergent evolution (Lui et al., 2020). Tricin is found as an external, pendent molecule, coupled via a β-O-4 bond with S and G monolignols. Biomimetic oxidation assays and reaction efficiency assays of acidolysis, thioacidolysis, and derivation followed by reductive cleavage (DFRC) have led to the hypothesis that tricin may serve as a nucleation site for grass lignin polymerization (Lu and Ralph, 1997; Lan et al., 2015; Lan et al., 2016b). Tricin is derived from the phenylpropanoid intermediate *p*CA-CoA, generated by 4CL during early in the monolignol biosynthesis pathway (Jiang et al., 2016) (**Figure 1**). *p*CA-CoA is shunted out of phenylpropanoid metabolism and committed to flavonoid biosynthesis via chalcone synthase (CHS) (Eloy et al., 2017; Lam et al., 2022). Naringenin chalcone is then isomerized to naringenin via chalcone isomerase (CHI) (Tetreault et al., 2021), and flavone synthase (FNS) catalyzes conversion of naringenin to flavone biosynthesis.

Two types of FNS have been characterized: FNS type II (*FNSII*), which appears to be present in all plants; and FNS type I (*FNSI*), which has thus far only been found co-expressed with *FNSII* in *O. sativa* (Lee et al., 2008; Lam et al., 2014), *Z. mays* (Righini et al., 2019), and in most of the *Apiaceae* family (Gebhardt et al., 2005, 2007). Metabolic and histochemical analysis of an *osfnsII* knockout and *osfnsI* T-DNA lines suggest that FNSII is the primary FNS involved in grass flavone biosynthesis (Lam et al., 2014, 2017). Tricin synthesis continues with apigenin 3’-hydroxylase/chrysoeriol 5’-hydroxylase (A3’H/C5’H) hydroxylation of the C3 carbon on apigenin, forming luteolin (Lam et al., 2015). Surprisingly, subsequent methylation of the 3’ alcohol of luteolin has been reported to be catalyzed by the phenylpropanoid enzyme COMT in *Z. mays* (Fornale et al., 2017), *S. bicolor* (Eudes et al., 2017 *)* and *O. sativa* (Lam et al., 2019), generating chrysoeriol. Chrysoeriol is then hydroxylated at the C5 carbon by A3’H/C5’H to generate selgin, which is again methylated by COMT, generating tricin (Eudes et al., 2017; Fornale et al., 2017; Lam et al., 2019). Because tricin appears to be present in all tested monocots to date, future grass lignin characterizations should include flavone analysis to investigate how tricin fits into the grass lignin model, such as how its absence may affect saccharification, and how it contributes to overall grass fitness.

### 2.2 Grass monolignol translocation and polymerization

#### 2.2.1 Monolignol translocation models are likely conserved among gymnosperms, monocots, and dicots

**Figure 2:**
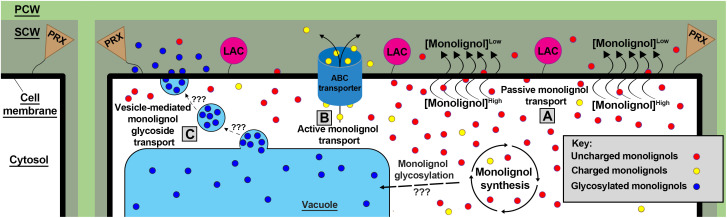
Three proposed models of monolignol transport. **(A)** Small uncharged monolignols (red) are theorized to passively diffuse through the plasma membrane over a concentration gradient driven by LAC and PRX monolignol polymerization. **(B)** Charged/conjugated (yellow) monolignols likely require active transport via ABC transporters to translocate across the plasma membrane lipid bilayer. **(C)** monolignols are glycosylated (blue) by an unknown mechanism and location in grasses. These glycosylated monolignols have been found stored in the vacuole and transported via an unknown mechanism to the plasma membrane via vesicle bodies. Once in the apoplast, membrane-bound LACs and PRXs polymerize monolignols via oxidative radical coupling. PCW, Primary Cell Wall; SCW, Secondary Cell Wall; LACs, laccases; PRXs, peroxidases.

Once synthesized, grass lignin monomers and conjugates are transported into the apoplast, likely via three mechanisms. Lignin monomer transport models such as passive diffusion (Vermaas et al., 2019b), active transport via ATP-binding cassette transporters (Miao and Liu, 2010; Alejandro et al., 2012), and vesicle storage and transport (Le Roy et al., 2016) have all been proposed to account for the highly variable properties of monolignols and monolignol conjugates and their transport across the cell membrane (del Río et al., 2022) (**Figure 2**). Although most monolignol transport research has been performed in *A. thaliana*, chemical properties of monolignols and the proposed biological roles of each transport mechanism suggest that all three models likely function in grasses (Dixon and Barros, 2019).

Passive diffusion of monolignols across the plasma membrane is supported by both modeling and experiments. Simulations found that hydrophobic “lignin-related compounds” are capable of diffusing through a homogeneous eukaryotic membrane, *Z. mays* root cell membranes, and gram-negative bacterial membranes (Vermaas et al., 2019b). Previous modeling corroborates these findings by observing “lignin-like” molecules transgressing a *Z. mays* cell lipid bilayer (Boija et al., 2007). Furthermore, a recent study performed in *A. thaliana* observed monolignols accumulating intracellularly when extracellular lignin polymerization was inhibited, suggesting that lignin polymerization generates a concentration gradient that drives passive monolignol diffusion into the apoplast (Perkins et al., 2022). The passive diffusion model is biomechanically simple, and is likely the dominant mechanism of monolignol translocation into the apoplast.

However, modeling experiments suggest some monolignols and glycosylated monolignol conjugates are physically incapable of passively diffusing through lipid bilayers (Vermaas et al., 2019b), and thus require active transport mechanisms to transit into the apoplast. A pair of studies found *A. thaliana* ATP binding cassette (ABC) transporter genes *AtABCG11*, *AtABCG22*, *AtABCG29*, and *ABCG36* were differentially co-expressed with a phenylpropanoid synthesis transcription factor *AtMYB58* during cell wall development in stems and roots (Takeuchi et al., 2018a, b). These findings are consistent with an earlier study of *A. thaliana* AtABCG29, which when expressed in yeast, was observed to actively transport H monomers across yeast cell membranes (Alejandro et al., 2012). The *Atabcg29* lines reportedly lack a significant lignin-deficient phenotype but show a decreased root growth phenotype and slight decreases in H, G, S monomers, flavonols, and glucosinolates. The lack of a significant lignin-deficient phenotype in singular lignin transporter mutations and concomitant considerable presence of lignin and glucosinolates in *Atabcg29* cell walls suggest that multiple transporters may be involved in actively translocating hydrophilic monolignols and monolignol conjugates, and that gradient-driven hydrophobic monolignol passive diffusion works in parallel with active transport.

Vesicle-mediated monolignol, flavonoid, and other aromatic molecule storage and subsequent deployment may be facilitated glucoside conjugation (Zhao, 2015; Perkins et al., 2019). Monolignol glucosides are synthesized by UDP-glycosyltransferases, and are a putative storage configuration of monolignols (Dima et al., 2015). Glycosylated monolignols are found in vacuoles of *A. thaliana* leaf cells (Dima et al., 2015), and are transported into the apoplast during cell lignification in a gymnosperm (Vaisanen et al., 2020), dicots (Morikawa et al., 2010; Tsuyama et al., 2013, 2019), and recently in the monocot *Phyllostachys pubescens* (Shimada et al., 2021). More research is needed to determine if cereals deploy sequestered monolignol glucosides after injury or pathogen detection; a mechanism not yet supported by genetic evidence (Jeon et al., 2022). Furthermore, the magnitude by which grass monolignols and glycosylated monolignol conjugates are actively transported/translocated to the apoplast through passive diffusion, active transport, or vesicle-mediated exocytosis remains uncertain and requires further investigation.

#### 2.2.2 Grass monolignol polymerization is driven by oxidative radical coupling

Once in the apoplast, mobile monolignols undergo polymerization via laccases (LACs) and peroxidases (PRXs) (Zhao et al., 2013; Schuetz et al., 2014; Francoz et al., 2015; Moural et al., 2017; Tobimatsu and Schuetz, 2019). LACs and PRXs facilitate a free radical oxidative coupling reaction, responsible for the combination of lignin monomers and oligomers with greater-order lignin polymer fragments (Ralph et al., 2004b). Radical coupling is capable of generating certain bond configurations of lignin polymers due to the preferential stability of free radical monolignol resonance structures (Ralph, 2010; Ralph et al., 2019). Studies of lignin polymer bonding configurations analyzed in gymnosperms, monocots, and dicots have found the most stable monolignol resonance structures facilitate a high probability of β-O-4, β-β, and β-5 bond formation (Ralph et al., 2004b; Sangha et al., 2012; Kim et al., 2023).

Lignin polymer compositions and purported stereospecificity have been proposed to be templated by dirigent proteins (Davin et al., 1997; Burlat et al., 2001; Davin and Lewis, 2005). However, the role dirigent proteins contribute to lignin polymerization remains somewhat controversial (Hatfield and Vermerris, 2001; Ralph et al., 2008; Paniagua et al., 2017), as lignin polymers are optically inactive (Ralph et al., 1999) and genetic evidence of dirigent proteins orchestrating an optically active lignin polymer is lacking (Ralph et al., 2008). There is evidence that a family of dirigent proteins, such as Enhanced Suberin 1 (ESB1) and dirigent proteins DIR9, DIR16, DIR18, DIR24, and DIR25, which coordinate lignin polymerization during Casparian strip biogenesis (Hosmani et al., 2013; Wang et al., 2019; Wang et al., 2022c; Gao et al., 2023). These findings provide insights into potential connections between lignan and lignin biosynthesis, and support functions of dirigent proteins in cell wall development and in response to stress (Paniagua et al., 2017).

#### 2.2.3 Grass lignin polymerization is tentatively modeled after *A. thaliana* LAC and PRX functional analysis

The mechanisms that govern lignin polymerization in grasses are largely uncharacterized. However, lignin polymerization studies in *A. thaliana* suggest that the mechanisms that control *A. thaliana* lignin deposition may be conserved in grasses. For example, “the good neighbor hypothesis” describes xylem cell lignification as non-cell autonomous (Smith et al., 2013; Smith et al., 2017b). Although the good neighbor hypothesis has thus far only been tested in *A. thaliana*, apoptotic grass xylem cells cannot autonomously lignify, suggesting that grass xylem cell lignification is also non-cell autonomous.

Apoplastic LACs utilize copper to generate reactive oxygen species (ROS) from molecular oxygen. These ROS are subsequently employed in the combinatorial coupling of monolignols and oligomers with greater-order lignin polymer fragments (Ralph et al., 2004b). LACs are currently thought to significantly contribute to vascular bundle lignification in *A. thaliana* because a *lac4lac11lac17* mutant displayed poor vascular lignification, significantly narrower root diameter, indehiscent anthers, and a severe negative growth phenotype (Zhao et al., 2013). The same study hypothesized that PRX activity is not redundant to LAC activity, as PRX activity was unable to compensate for the triple *lac* mutant. In a later study, fluorescently labeled AtLAC4 was found immobile once excreted throughout the secondary cell walls of xylem vessels, xylem fibers, and interfascicular fibers, supporting the hypothesis that *A. thaliana* LAC secretion and activity broadly governs lignification in vascular bundles and interfascicular fibers (Chou et al., 2018). A more recent study of quadruple and quintuple *A. thaliana lac* mutants found five *A. thaliana LAC* paralogs to be nonredundant, to lignify specific tracheary and structural cell types, and show different affinities for monolignols (Blaschek et al., 2022).

PRXs generate ROS from H_2_O_2_ that also facilitate lignin combinatorial coupling (Ralph et al., 2004b). Observations of LAC and PRX co-expression in lignifying tissues has generated debate over how apoplastic PRXs contribute a non-redundant role to lignin polymerization (Barros et al., 2015). The first substantial clue of PRX nonredundant function was found after Casparian strip (CS) development in *A. thaliana* was severely delayed due to induced mutation of endodermis-specific *prx64* (Lee et al., 2013). Supporting this finding, the *A. thaliana lac4lac11lac17* triple mutant (Zhao et al., 2013) was observed to have a lignified and functional CS, suggesting that either a CS-specific *LAC* homolog was still active, or that LACs do not significantly contribute to CS lignification. Later, fluorescently labeled AtPRX64 was observed to localize in the middle lamella and cell corners of interfascicular fibers, whereas AtLAC4 did not (Chou et al., 2018), suggesting that PRX significantly contributes to cell corner lignification. Recently, PRXs were found to be required and nonredundant for CS lignification after a quintuple *A. thaliana prx* mutant showed no detectable lignin in the CS, whereas a nonuple *lac* mutant showed no changes in CS integrity or lignification (Rojas-Murcia et al., 2020). To our knowledge, simultaneous disruption of *LAC* and *PRX* genes in grasses has not been studied.

The increasing availability of high-quality genomic databases combined with foundational models established in *A. thaliana* suggest a promising future to examine grass LAC and PRX function. For instance, *LAC* genes have been characterized in *T. aestivum* (Zhong et al., 2023), *O. sativa* (Liu et al., 2017), and *S. bicolor* (Wang et al., 2017), whereas PRXs have been identified in *B. distachyon* (Zhu et al., 2019), *P. virgatum* (Moural et al., 2017), and *Setaria viridis* (Simoes et al., 2023). Similar to *A. thaliana*, both BdLAC5 and BdLAC6 (orthologs to *AtLAC17* and *AtLAC4*, respectively) were observed to localize in the apoplast near tracheary element secondary cell walls (Wang et al., 2015b). In addition, the *B. distachyon LAC* double mutant (*lac5lac8*) showed a 20-30% decrease in interfascicular fiber lignin, but only a mild growth defect (Le Bris et al., 2019).

Although these data in grasses are somewhat analogous to LAC studies in *A. thaliana*, the high concentrations of covalently bound hydroxycinnamic acids and hydroxycinnamic acid conjugates found in grass secondary cell walls may present an additional layer of complexity to the mechanisms that facilitate grass lignin polymerization (Hatfield and Marita, 2010). Recent genetic and metabolomic evidence generated in *S. viridis*, *B. distachyon*, and *Saccharum* spp. suggest that BAHD acyltransferases may significantly contribute to feruloylation of AX (de Souza et al., 2018, 2019). How grass LACs interact with FA is unclear, as the *Bdlac5* mutant in *B. distachyon* displayed a 40% increase in esterified (cell wall-bound) FA and a 15% decrease in ether-bound (protein or lignin bound) FA (Wang et al., 2015b). Likewise, the role of grass PRXs in relation to hydroxycinnamic acid polymerization is largely unknown. A seminal study with *Z. mays* cell cultures observed that 60% of FA dimerized with dilute H_2_O_2_ addition to the cell suspension, suggesting that expression of PRX during early secondary cell wall development may also mediate FA acylation to AX (Grabber et al., 2000; Ralph et al., 2004a). These data, and lack thereof, emphasize there is still much to learn about the specific molecular mechanisms governing grass lignin polymerization, and that not all grass LAC and PRX functions can be simply extrapolated from *A. thaliana* studies.

## 3 Biological roles of lignin in grasses

### 3.1 The role of lignin in stem strength, drought, and heavy metal stress tolerance

#### 3.1.1 diFA-mediated cell wall crosslinking significantly contributes to grass stem mechanical strength

Lignified grass tissue has been reported to facilitate proper development and improve abiotic stress resistance (**Table 1**). Lignin compositions are variable between and amongst species, tissue types, and even cell wall layers (Vanholme et al., 2010; Zeng et al., 2014; Menard et al., 2022). These lignin compositions are often quantified as a simple ratio measurement of total S monomers to total G monomers, but many past analyses of grass lignocellulose compositions lack quantification of cell wall-bound and ether-linked hydroxycinnamic acids. Future inclusion of this data is significant to grass lignocellulose analysis because recent studies suggest decreased S/G ratios combined with varying cell wall-bound hydroxycinnamic acid content appear to enhance mechanical strength of the load-bearing interfascicular and xylary fibers, tracheary elements, and sclerenchyma cells (Le Bris et al., 2019; Li et al., 2022a).

**Table 1:** A summary of stem lodging, heavy metal, and drought stress response studies that document changes in lignin-related gene expression and lignin content in grasses.

Species	Tissue	Transcriptional response	Phenolic profile change	Stress resistance	Citation	
*H. vulgare*	Stem	*↑PTAL, ↑PAL, ↑CCR, ↑COMT, ↑LAC, ↑PRX*	↑FA, ↑CA, ↑*p*CA, ↑Total lignin	Stem lodging	(Yu et al., 2021)
	Stem	*↑PAL*	↓Total phenolics	Stem lodging	(Begovic et al., 2018)
	Leaf, Root	*↓PAL, ↓CCoAOMT*	↑*p*CA, ↑FA	Drought	(Chmielewska et al., 2016)
*O. sativa*	Stem	*-*	↑Total lignin	Stem lodging	(Gui et al., 2018)
	Root	*↑CCoAOMT, ↑COMT*, *↑PRX, ↑LAC*	↑Total lignin	Fe	(Stein et al., 2019)
	Root	*↑PAL, ↑C4H, ↑C3H, ↑4CL, ↑CCoAOMT, ↑CCR, ↑F5H, ↑COMT, ↑LAC, ↑PRX*	↑Total lignin	Cu	(Liu et al., 2015)
	Root	–	↑Total lignin	Drought	(Ouyang et al., 2020)
*P. virgatum*	Leaf, Stem, Root	*↑LAC*	↑Total lignin	Cu, Fe, Ni, Cd	(Li et al., 2022b)
*S. cereale*	Stem	–	↑Total lignin	Stem lodging	(Muszynska et al., 2021)
*T. aestivum*	Stem	–	↑H, ↓S/G	Stem lodging	(Muhammad et al., 2020)
	Stem	*↑CAD*	–	Stem lodging	(Ma, 2010)
	Stem	–	↑H, ↓S/G, ↑Total lignin	Stem lodging	(Zheng et al., 2017)
	Stem	–	↑Total lignin	Stem lodging	(Bisht et al., 2022)
	Leaf, Root	*↑PAL, ↑4CL, ↑CAD, ↑LAC*	↑Total lignin	Pb, Cu	(Li et al., 2023)
	Root	*↑PAL, ↑TAL, ↑PRX*	↑Total phenolics, ↑Total flavonoids	Pb, Cu	(Janczak-Pieniazek et al., 2023)
	Root	*↑CAD, ↑Flavonoid CYP450s, ↓PAL, ↓AUX, ↓PRX*	–	Drought	(Chaichi et al., 2019)
*Z. mays*	Leaf	–	↓CAF, ↓FA, ↑Total phenolics	Cu, Cd, Pb	(Kisa et al., 2016)
	Stem	–	↑H, ↓S/G, ↑FA, ↑*p*CA	Stem lodging	(Manga-Robles et al., 2021)
	Root	*↑CCR1*	↑Total lignin	Drought	(Fan et al., 2006)

Studies are organized based on species, tissue examined, transcriptional response, change in phenolic profile, stress tolerance and citations are provided. See legends of **Figures 1**, **2** for definitions of the abbreviations of monolignols and phenylpropanoid biosynthesis genes. CA, Cinnamic acid; *p*CA, *p*-Coumaric acid; FA, Ferulic acid; Total phenolics, total cell wall-bound and soluble phenolics; Total flavonoids, total soluble flavonoids; H, *p*-Coumaryl alcohol; G, Coniferyl alcohol; S, Sinapyl alcohol. Those responses with (-) denote that the change was not determined in the study. “↑”, increasing; “↓”, decreasing.

Increased diFA-mediated crosslinking characteristic of monocots significantly contributes to resistance to mechanical stressors (Ralph, 2010; Ralph et al., 2019; Eugene et al., 2020). Consistent with this, decreased S/G ratios and increased diFA content are positively correlated with culm stability and overall plant height (Cesarino, 2019; Xue et al., 2020; Manga-Robles et al., 2021; Li et al., 2022a) (**Table 1**). Biochemical and phenotypic analysis of lodging resistant *Z. mays* inbred lines were found to have decreased S/G ratios and significantly increased cell wall FA, diFA, *p*CA, and AX (Manga-Robles et al., 2021). Similarly, lodging-resistant *T. aestivum* lines revealed that a 16.9% reduction in S/G ratio, increased size and number of large vascular bundles, and reduced number of small vascular bundles was associated with increased culm strength (Muhammad et al., 2020). Lodging-resistant *H. vulgare* lines have significantly decreased culm S/G ratio and a significant increase in cell wall-bound FA and *p*CA compared to lodging susceptible lines (Yu et al., 2021). Finally, a recent analysis of a RNAi knockdown of *f5h* in *H. vulgare* found a significant decrease in S/G ratios, cell wall-bound *p*CA, and cell wall-bound FA, but no change in total lignin content or stem mechanical strength (Shafiei et al., 2023). Taken together, these findings offer evidence to suggest reduced S/G ratios and increased diFA-mediated crosslinking contribute to grass culm mechanical strength and flexibility, and that significant targeted reductions in S monomer content do not significantly compromise grass stem structural integrity.

Although there appears to be a clear correlation between grass cell wall crosslinking and overall stem mechanical strength, the extent to which total lignin deposition contributes to stem strength is still unclear. For instance, studies in *O. sativa* (Gui et al., 2018), *H. vulgare* (Begovic et al., 2018), and *T. aestivum* (Bisht et al., 2022) found total culm lignin content to be positively correlated with increased culm strength. Conversely, no correlation between total stem lignin and stem strength were found in population-scale studies of *S. italica* (Tian et al., 2015), *Avena sativa* (Argenta et al., 2022), *Z. mays* (Manga-Robles et al., 2021), and *T. aestivum* (Muhammad et al., 2020). These inconsistencies in data may be resolved in future grass lignocellulose analysis by prioritizing more comprehensive cell wall phenolic analyses that include quantification of S/G ratio with cell wall-bound FA, *p*CA, and tricin. Inclusion of these data with parallel qualitative histochemical assays of structurally significant anatomical features will provide a more holistic analysis of how degrees of cell wall crosslinking, rather than discrete monolignol content, contributes to grass stem strength.

#### 3.1.2 Can drought-induced abscisic acid signaling enhance grass root xylem lignification and improve drought stress resistance?

Drought stress is managed by plants via both avoidance and tolerance strategies. Drought avoidance employs one or more physiologic changes in metabolism or anatomy to mitigate water loss or improve water uptake, and is initiated by drought sensing in roots via a complex series of ROS/Ca^2+^-mediated signaling pathways that induce changes in abscisic acid (ABA) and auxin production and transport (Zhu, 2016; Kalra et al., 2023). Some of these signals elicit subterranean drought avoidance growth responses such as increased root length, increased root penetration angle, and increased lateral root length—all necessary for plants to access soil moisture at depth (Vadez, 2014; Chaichi et al., 2019; Lamb et al., 2022). On the other hand, less plastic drought tolerance strategies involve innate phenotypes, such as photosynthetic anatomy and leaf shape that contribute to enduring prolonged drought stress. Lignification of secondary cell walls in tracheary elements can be classified as a drought tolerance phenotype, as xylary lignification imbues tracheary elements with hydrophobicity while providing structural reinforcement to resist cavitation caused by negative pressure generated during evapotranspiration (Fan et al., 2006; Chmielewska et al., 2016).

**Figure 3:**
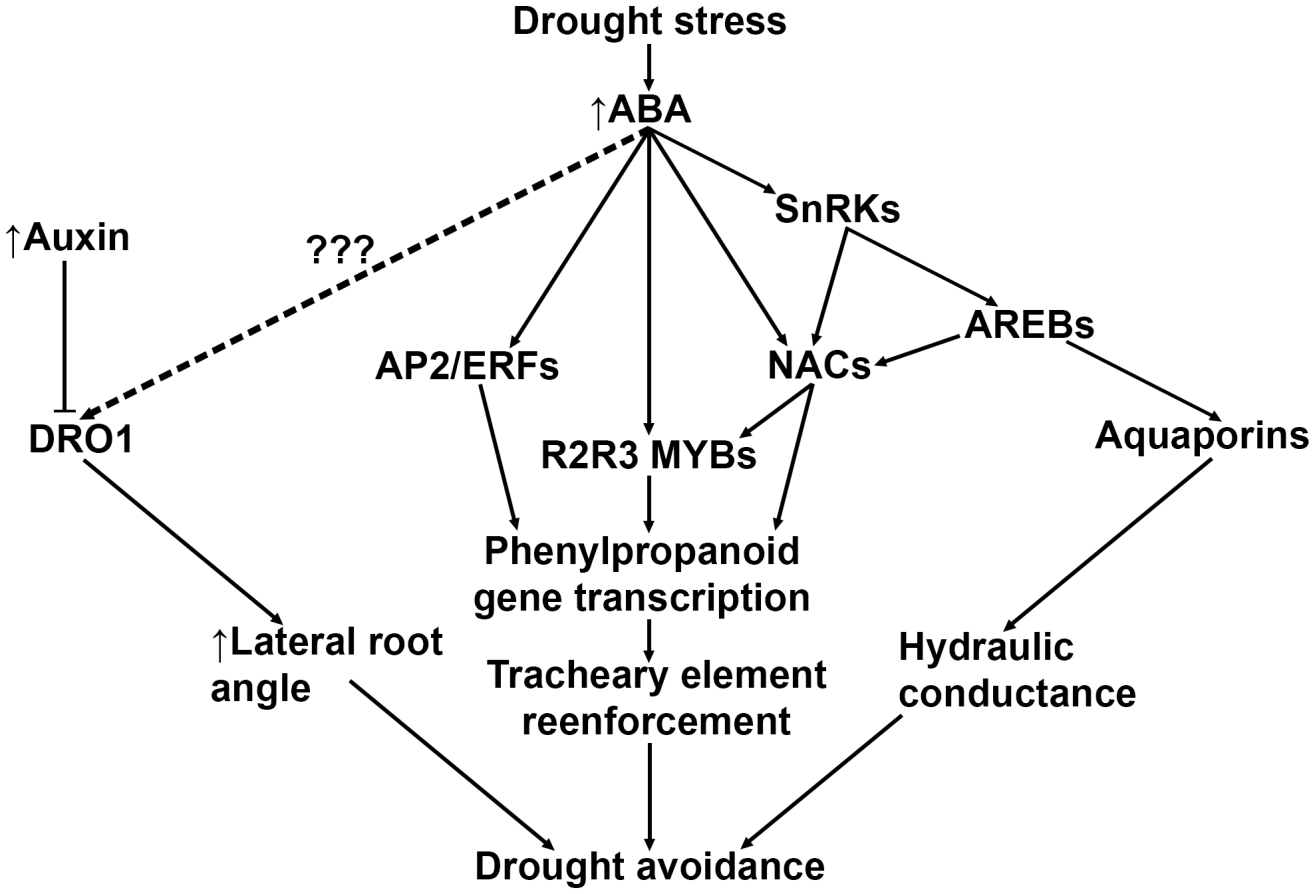
A general model depicting known and predicted drought-induced ABA signaling pathways that result in drought tolerance mechanisms. Drought-induced ABA upregulates *DRO1*, but the intermediary signaling mechanism is currently unknown. ABA also directly and indirectly upregulates several higher order transcription factors such as *AP2/ERFs* and *NACs*, which both directly and indirectly modulate phenylpropanoid gene transcription. Lastly, drought-induced ABA is responsible for upregulating transcription of aquaporins in roots, which appear to be coregulated with genes in the phenylpropanoid biosynthetic pathway. ABA, abscisic acid; *DRO1, DEEP ROOT 1*; SnRK, Sucrose nonfermenting1-related Kinases; AREBs, Abscisic Acid-Responsive Element Binding proteins; NACs, NAM-ATAF-CUC2 transcription factors; AP2/ERFs, APETALA2/Ethylene-Responsive Factors.

Recent advances in understanding the signaling pathways that govern drought stress suggest that avoidance and tolerance strategies may overlap with root lignification. A previous study proposed to reduce xylem diameter of *T. aestivum* seminal roots as a means to improve yield (Richards and Passioura, 1989). The authors hypothesized that the reduced seminal root xylem diameter may lower water conductance and slow the depletion of soil moisture, thereby preserving water during anthesis when plants are most sensitive to drought stress (Fageria, 1992). In support of this hypothesis, drought-adapted *O. sativa* lines showed increased root stele lignification and decreased root diameter after drought treatment (Hazman and Brown, 2018). Drought avoidance root architectural modifications are likely mediated by ABA signaling (Uga et al., 2013; Singh and Laxmi, 2015; Xu et al., 2020; Feng et al., 2022) and partially modulated by endogenous root auxin transport (Uga et al., 2013; Yu et al., 2016) (**Figure 3**). For example, ABA induces the expression of the grass-specific gene *DEEP ROOT 1* (*DRO1*), which increases root growth angle in the root elongation zone in *O. sativa* (Arai-Sanoh et al., 2014) and *Z. mays* (Feng et al., 2022). A positive correlation between increased root angle and drought tolerance has also been reported in *Z. mays* (Ali et al., 2015) and *S. bicolor* (Djanaguiraman et al., 2023), suggesting that an uncharacterized ABA-induced activation of *DRO1* may also induce lignification in roots.

ABA was recently observed to release the constitutive inhibition of Snf1 (Sucrose nonfermenting1)-Related Kinase 2 (SnRK2) in *A. thaliana*, inducing secondary cell wall remodeling and lignin deposition via phosphorylation of SECONDARY WALL THICKENING PROMOTING FACTOR 1 (NST1) (Liu et al., 2021a). NST transcription factors are part of the NAM-ATAF-CUC2 (NAC) family of master regulators and form part of a highly conserved multi-tiered transcription factor cascade in *A. thaliana* that controls activation of the phenylpropanoid biosynthesis pathway (Singh and Laxmi, 2015; Ohtani and Demura, 2019; Miyamoto et al., 2020; Hussey, 2022). NST1 binds to promoters of numerous downstream MYB transcription factors that directly regulate cell wall biogenesis and monolignol enzymes in both *A. thaliana* (Singh and Laxmi, 2015; Liu et al., 2021a) and grasses (Zhao and Bartley, 2014; Velez-Bermudez et al., 2015; Miyamoto et al., 2020). Both *O. sativa* and *Z. mays* NAC transcription factors directly bind to *MYB46/83* promoter regions, which modulate secondary cell wall biosynthesis (Zhong et al., 2011). Similarly, overexpression of transcription factor *SiMYB56* in *O. sativa* resulted in increased ABA synthesis, increased lignin deposition in leaves, stems, and roots, and improved overall drought tolerance (Xu et al., 2020).

ABA, jasmonic acid (JA), and salicylic acid (SA) also directly activate *NAC* promoter elements and modulate lignin biosynthesis independent of SnRK2 signaling (Nakashima et al., 2014; Singh and Laxmi, 2015). For example, ABA, drought, and NaCl treatment and overexpression *OsNAC5* were found to upregulate *OsCCR10* in drought-resistant *O. sativa* roots (Bang et al., 2022). In the same study, overexpression of *OsNAC5* and OsCCR10 was correlated with improved drought tolerance and significantly increased root lignin content. Similarly, transgenic *O. sativa* overexpressing *OsNAC17* showed increased expression of *PAL*, *CCR*, *CAD*, and *PRX* genes leading to significant lignin accumulation in leaf and root tissues (Jung et al., 2022).

APETALA2/ethylene-responsive factor (AP2/ERF) transcription factors also mediate drought-induced ABA signaling via activation by DREB transcription factors, resulting in drought tolerance in *O. sativa* (Liu et al., 2012; Wu et al., 2022; Xie et al., 2022). Treating *O. sativa* leaves and roots with ABA resulted in upregulation of OsERF83, increase in vascular lignification, enhanced LAC activity, elevated *CAD* expression, and improved drought tolerance (Jung et al., 2021). Additionally, an ABA-responsive AP2/ERF transcription factor OsERF71 was observed to bind directly to the *OsCCR1* promoter (Lee et al., 2016). The same study found overexpression of *OsERF71* was correlated with a significant increase in root diameter and a significant increase in *PAL, C4H, CCR*, and *CAD* transcription. Taken together, these data suggest that ABA induces multiple drought avoidance responses that modify root architecture, with some of these ABA signaling pathways include upregulation of monolignol synthesis genes and subsequent increased root lignin deposition. How increased root lignin deposition contributes to drought tolerance and/or avoidance and how ABA directly modulates root lignin deposition is still unclear and requires further testing. Elucidation of these ABA signaling pathways in grass roots will likely lead to characterization novel breeding targets that address the need of more drought-tolerant crops.

#### 3.1.3 The lignified and suberized Casparian strip creates an ionic barrier

The transport of both water and ions into the root vasculature is regulated at the CS, the hydrophobic barrier of the root endodermis found in all terrestrial plants (Naseer et al., 2012; Doblas et al., 2017). The hydrophobicity of the CS is attributed to an asymmetric deposition of both lignin and suberin (Doblas et al., 2017; Calvo-Polanco et al., 2021); with low S/G ratio lignin primarily fortifying cell wall junctions, while suberin primarily fortifies the middle lamella (Naseer et al., 2012; Reyt et al., 2021). Recent studies in *A. thaliana* and *O. sativa* have observed Casparian Strip Membrane Domain Proteins (CASPs) to initiate CS formation in cell corners, defined by the extracellular protease LORD OF THE RINGS1 excreted by neighboring cells (Kolbeck et al., 2022; Yang et al., 2022; Barbosa et al., 2023). CASPs proceed to direct cell junction lignification via recruitment of the dirigent-domain containing protein Enhanced Suberin1 (ESB1), PRX, and NADPH oxidase F in *A. thaliana* (Hosmani et al., 2013; Gao et al., 2023), *Z. mays* (Wang et al., 2022c), and *O. sativa* (Wang et al., 2019, 2020). A CS hormone/kinase monitoring system characterized in *A. thaliana* called the Schengen pathway seals and maintains the CS ring utilizing Casparian Strip Integrity Factors (CIFs), GASSHO1 (GSO1)/SCHENGEN3 (SNG3) kinase receptors, PRXs, and a family of dirigent proteins (Doblas et al., 2017; Nakayama et al., 2017; Fujita et al., 2020). Recently, *O. sativa CIF1* and *SNG3* orthologs were functionally characterized (Zhang et al., 2023a), suggesting grasses possess a similar pathway.

ABA and ethylene antagonistically modulate suberization and lignification of the endodermis to regulate the bidirectional flow of ions and water (Doblas et al., 2017). Surprisingly, two independent studies of *A. thaliana* Schengen pathway higher order mutants found that disruption of lignification and suberization of the CS did not significantly change hydraulic conductance (Calvo-Polanco et al., 2021; Reyt et al., 2021). Instead, both studies independently concluded that aquaporin expression significantly influences hydraulic conductivity. The *A. thaliana* Schengen pathway higher order mutants displayed compositionally different lignin in the endodermal cell corners (H: 19%, G: 79%, S: 2%) compared to lignin polymerized in endodermal cell junctions (H:5%, G:87%, S:8%) (Reyt et al., 2021), but the function of this compositional difference is unclear.

Unique metabolic aspects of grass lignin metabolism may be strategically leveraged to quantify grass lignin CS compositions, investigate if lignin compositions in grass CS are a product of selective PRX-mediated polymerization, and test if these compositional differences between cell corners and endodermal junctions contribute to ion permeability. For instance, membrane-bound PRXs have been attributed to the lignification of *A. thaliana* endodermal cell corners (Rojas-Murcia et al., 2020). Functional characterization of a *AtPRX64* ortholog in grasses is first needed to test if CS lignification is also mediated by one or more PRXs. Furthermore, *Z. mays* PRXs isolated from cell suspension cultures were found to be inefficient at oxidizing S monomers, and were suggested to oxidize S monomers more efficiently through *p*CA-S via radical transfer (Hatfield et al., 2008). Perturbing PMT function in a grass CS-specific *prx* mutant background and analyzing its phenolic composition may test if PRX employs *p*CA-S to facilitate radical transfer and subsequent polymerization of S monomers into the CS (Hatfield and Marita, 2010). Lastly, measuring the lignin composition and changes in CS ion permeability of a grass *lac pmt* mutant versus a *prx pmt* mutant may provide insight into the functional significance of differential lignin compositions of cell corners and endodermal junctions, as previously described in *A. thaliana* Schengen pathway mutants (Reyt et al., 2021).

#### 3.1.4 LAC activity likely contributes to heavy metal ion sequestration in grass secondary cell walls

Research into heavy metal tolerance is increasing as major cereal crops are experiencing significant yield loss due to irrigation containing zinc (Zn), ferrous iron (Fe), nickel (Ni), copper (Cu), cadmium (Cd), chromium (Cr), and lead (Pb) from industrial runoff (Mir et al., 2021; Riyazuddin et al., 2022; Skuza et al., 2022; Li et al., 2022b; Xu et al., 2023). Mounting evidence suggests cell wall thickening of both woody and herbaceous plants contributes a partial but significant exclusion barrier where heavy metals are sequestered via ion exchange and chelation with methyl-esterified pectins, phenolic acids, organic acids, and ROS (Krzeslowska, 2011; Parrotta et al., 2015; Berni et al., 2019; Gao et al., 2021; Sytar et al., 2021) (**Table 1**). Both the physical barrier created by polysaccharides, callose, and lignin and ROS-mediated chelation of heavy metals into the cell wall are theorized to significantly reduce the rate of heavy metal uptake (Krzeslowska, 2011; Angulo-Bejarano et al., 2021).

LAC activity may significantly contribute to improved heavy metal tolerance by facilitating ROS-mediated chelation of heavy metals into the lignocellulose matrix. High Cu exposure led to significant upregulation of lignin biosynthesis genes, increased lignin deposition and LAC and PRX activity in *O. sativa* roots (Liu et al., 2015). Exposure to toxic concentrations of Cu, Pb, and Cd triggered a lignin biosynthesis and polymerization response in roots and significantly reduced soluble CAF and FA in *Z. mays* leaves (Kisa et al., 2016). This same study also demonstrated that stress responses to heavy metal exposure may not be localized only to the treated tissue. Recent studies in *P. virgatum* (Li et al., 2022b) and *T. aestivum* (Janczak-Pieniazek et al., 2023; Li et al., 2023) report similar findings of systemic increased monolignol biosynthesis, LAC activity, and lignin deposition in response to heavy metal exposure.

Thus, heavy metal exposure appears to elicit a systemic increase in lignification in grasses, but the reason behind this response is still not yet clear. Furthermore, the significant reduction of soluble CAF and FA reported in *Z. mays* leaves after Cu, Pb, and Cd root treatments (Kisa et al., 2016) are curious. Perhaps increased monolignol synthesis and LAC activity may lower CAF and FA pools during the stress response. Specific LAC isoforms may also facilitate the hypothesized ROS-mediated chelation of heavy metals, as several non-discrete combinations of *O. sativa LACs* were found upregulated in response to Cu, Cd, Cr, and Pb exposure (Liu et al., 2017). In support of this postulation, two apoplastic *P. virgatum LACs* were recently found to be upregulated after Cu, Ni, Fe, and Cd treatments (Li et al., 2022b). Taken together, these studies highlight how model systems and tools developed in basic research can be applied to address modern agricultural problems. However, many questions remain unanswered, such as what role does PRX contribute to heavy metal stress-induced lignification, what compositional changes (if any) arise in lignin in response to heavy metal stress, and how these compositional changes may contribute to decreased heavy metal uptake?

### 3.2 Lignin and biotic stress resistance in grasses

#### 3.2.1 Do elevated S/G lignin ratios improve resistance of fungal pathogens?

**Figure 4:**
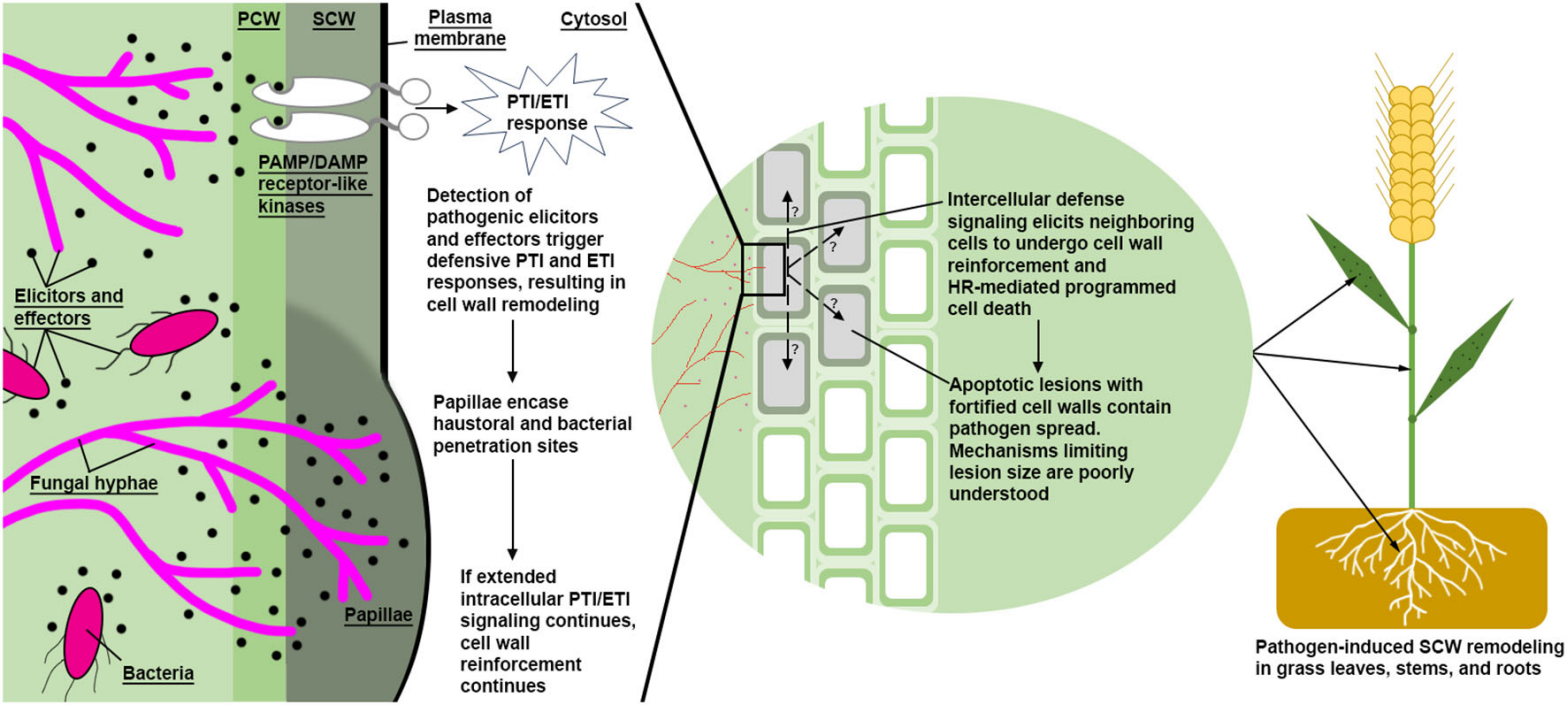
A simplified model of pathogen defense via induced SCW reinforcement. Bacterial and fungal hyphae (magenta colored) both produce PAMPs and DAMPs. Detection of these pathogen elicitors and effectors lead to reactive SCW reinforcement via synthesis and deposition of callose, lignin, arabinoxylans, pectin, ROS, and antimicrobial peptides. A proposed intercellular signal mechanism may transmit to neighboring cells, inducing SCW reinforcement and/or non-cell autonomous hypersensitive response, resulting in apoptotic lesions surrounding infection sites. High S/G ratio stress lignin has been characterized at the margins of lesions, which are thought to physically reinforce affected cells from further pathogen introgression. PCW, Primary Cell Wall; SCW, Secondary Cell Wall; HR, Hypersensitive Response; PTI, Pathogen-Triggered Immune; ETI, Effector-Triggered Immune.

In addition to abiotic stress, lignin has also been linked to biotic stress responses in grasses as summarized in **Table 2**. Types of fungal pathogenesis can be broadly described in two categories: biotrophs, which develop feeding sites in plant cells and extract nutrients from living plant tissue; and hemibiotrophs/necrotrophs, which secrete digestive cocktails and extract nutrients from the resulting pool of cellular debris (Doehlemann et al., 2017; Rajarammohan, 2021). Elicitation and signal transduction of pathogen triggered immune responses (PTI) and pathogen effector triggered immune responses (ETI) are complex (Yuan et al., 2021), and often lead to hypersensitive response (HR)-mediated cell death (Balint-Kurti, 2019; Guo and Cheng, 2022). One example of a host PTI/ETI response to biotrophic pathogens is a cell autonomous synthesis of papillae (**Figure 4**), which are hypothesized to slow or prevent biotrophic pathogenesis by reactively encasing the pathogen feeding organ in recalcitrant appositions (Israel et al., 1980; von Ropenack et al., 1998; An et al., 2006; Underwood, 2012; Bellincampi et al., 2014; Wang et al., 2021). Papillae, as seen in biotrophic *Blumeria graminis* f. sp. *Hordei* (powdery mildew) resistant *H. vulgare*, are thought to be generally enriched with increased β-1,3-glucan, callose, “stress lignin,” pectin, and AX (Chowdhury et al., 2014), in addition to antimicrobial peptides, ROS, and other antimicrobial metabolites (An et al., 2006; Underwood, 2012; Bellincampi et al., 2014; Wang et al., 2021). Necrotrophic pathogen PTI/ETI responses differ from biotrophic stress responses. Plants elicit a rapid but localized and controlled HR response at the infection site in response to necrotrophs and surround the affected tissue with “stress lignin” (Malik et al., 2020). Although the efficacy of HR-mediated programmed cell death has been questioned as an efficient defense strategy, an increased HR response combined with “stress lignin” deposition is believed to significantly inhibit pathogen spread and improve plant pathogen resistance (Ride, 1975; Balint-Kurti, 2019; Cesarino, 2019; Lee et al., 2019; Malik et al., 2020).

**Table 2:** A summary of biotic stress studies in monocot crops where differential lignin deposition and/or differential lignin synthesis-related gene expression was reported to significantly correlate with plant pathogen resistance.

Species	Tissue	Transcriptional responses and active genes	Phenolic profile change	Stress resistance	Citation
*H. vulgare*	Spike	*↑NAC, ↑ERF, ↑MYB, ↑PAL, ↑CHS, ↑4CL, ↑HCT, ↑CCR, ↑F5H, ↑CAD, ↑LAC, ↑PRX*	–	*Fusarium graminearum*	(Karre et al., 2017)
	Leaf	*PAL, CAD*	–	*Puccinia hordei, Blumeria graminis f.* sp. hordei	(Prats et al., 2007)
	Leaf	*PAL, CAD*	–	*Blumeria graminis f.* sp. hordei	(Kruger et al., 2002)
*Musa (spp.)*	Root	–	↑FA, ↑Total lignin	*Radopholus similis*	(Wuyts et al., 2007)
*O. glaberrima*	Root	*↑PAL, ↑CHS, ↑FNSII, ↑CCoAOMT, ↑CCR, ↑CAD*	–	*Meloidogyne graminicola*	(Petitot et al., 2017)
*O. sativa*	Leaf	*↓AP2/ERF, ↑PAL*	–	*Magnaporthe oryzae*	(Liu et al., 2012)
	Leaf	*↑PAL*	–	*Magnaporthe oryzae, Rhizoctonia solani*, *Xanthomonas oryzae pv.* oryzae	(Tonnessen et al., 2015)
	Root	–	↑Total lignin	*Meloidogyne graminicola*	(Galeng-Lawilao et al., 2019)
	Root	*↑PAL, ↑Callose synthase*	↑Total lignin	*Meloidogyne graminicola*	(Ji et al., 2015)
*S. bicolor*	Stem	*↑FNSII*	↑ Total flavonoids	*Spodoptera frugiperda*	(Grover et al., 2022b)
*T. aestivum*	Leaf	–	↑S	*Botrytis cinerea, Mycosphaerella pinodes*	(Ride, 1975)
	Leaf	–	↑S/G, ↑SA, ↑Total lignin	*Puccinia graminis f.* sp. tritici	(Menden et al., 2007)
	Leaf	*↑NAC, ↑MYB, ↑ABA responsive protein, ↑PAL, ↑CHS, ↑4CL, ↑COMT, ↑CAD, ↑LAC, ↑PRX*	–	*Fusarium pseudograminearum*	(Powell et al., 2017)
	Spike	–	↑S, ↑FA, ↑diFA	*Fusarium graminearum*	(Gunnaiah and Kushalappa, 2014)
	Spike	*↑PAL, ↑CHS, ↑COMT, ↑CAD, ↑Callose synthase, ↑LAC, ↑PRX*	–	*Fusarium graminearum*	(Dhokane et al., 2016)
	Stem	*↑COMT*	↑S	*Rhizoctonia cerealis*	(Wang et al., 2018)
	Stem	*↑CAD*	–	*Rhizoctonia cerealis*	(Rong et al., 2016)
	Root	*↑PAL, ↑HCT, ↑PRX*	–	*Heterodera avenae*	(Kong et al., 2015)
	Root	*-*	↑Total lignin	*Pratylenchus neglectus*	(Thompson et al., 2015)
*T. monococcum*	Leaf	*↑PAL, ↑CCoAOMT, ↑CCR, ↑F5H, ↑COMT, ↑CAD*	–	*Blumeria graminis f.* sp. tritici	(Bhuiyan et al., 2009)
*T. turgidum*	Spike	–	↑S/G	*Fusarium graminearum*	(Lionetti et al., 2015)
*Z. mays*	Stem	–	↑*p*CA, ↑S/G	*Sesamia nonagrioides*	(Gesteiro et al., 2021)
	Stem	–	↑diFA	*Sesamia nonagrioides*	(Barros-Rios et al., 2015)

Significant changes in cell wall phenolic profiles and gene expression profiles are denoted by arrow orientation. See legends of **Figures 1**-**3** for definitions of the abbreviations of monolignols and phenylpropanoid biosynthesis genes. *p*CA, *p*-Coumaric acid; FA, Ferulic acid; diFA, diferulic acid; SA, Sinapic acid; Total phenolics, total cell wall-bound and soluble phenolics; Total flavonoids, total soluble flavonoids; G, Coniferyl alcohol; S, Sinapyl alcohol. Those responses with (-) denote that the change was not determined in the study “↑”, increasing; “↓”, decreasing.

Deposition of distinct types of “stress lignin” in papillae and at the margins of lesions appear to be a common resistance strategy in response to both necrotrophic and biotrophic pathogens. Stress lignin was initially identified as an S-rich lignin through histochemical staining surrounding infection sites of necrotrophic fungus *Botrytis cinerea* and biotrophic fungus *Mycosphaerella pinodes* on *T. aestivum* leaves (Ride, 1975). Later research observed a 4-fold increase in soluble esterified sinapic acid and a S/G ratio increase from 0.55 to 1.43 in *T. aestivum* leaves seven days after inoculation with the biotroph *Puccinia graminis f.sp. tritici* (stripe rust) (Menden et al., 2007). Comparison of resistant and susceptible *Triticum turgidum* lines to *Fusarium graminearum* (Fusarium head blight) found that increased S/G ratios (0.121 in the resistant line versus 0.031 in the susceptible line) was a significant cell wall resistance factor (Lionetti et al., 2015). In line with this finding, an increase in *TaCOMT-3D* expression was found in two resistant *T. aestivum* lines 18 days after inoculation with *Rhizoctonia cerealis* (sharp eyespot disease) (Wang et al., 2018). The same study overexpressed and suppressed *TaCOMT-3D* in *T. aestivum* and found increased *TaCOMT-3D* expression was positively correlated with increased lignin deposition, particularly enriched with S monomers as determined by Mäule staining, and increased *R. cerealis* resistance. Taken together, the biosynthesis and localized deposition of chemically distinct stress lignin appears to enhance resistance against both biotrophic and necrotrophic pathogens (Lee et al., 2019).

A question then arises: why would grasses synthesize and polymerize a chemically distinct lignin in response to biotic stress? Recent advances in computational biology have enabled the simulation of small molecule interactions with cell wall carbohydrates, thus providing a method to test if increased S/G ratio lignin can enhance lignocellulose recalcitrance to enzymatic and chemical digestion. One such *in silico* simulation of lignin monomer, dimer, and polymer binding to a homogeneous cellulose surface found the free energies produced by the aqueous exclusion of the methoxy groups on S monomers and β–O–4 bound dimers likely promote the closest association of the lignin polymer with the cellulose matrix (Vermaas et al., 2019a). These data led to the hypothesis that increased S monomers and β–O–4 bound dimers likely contribute to an enhanced water exclusion mechanism by shielding cell wall carbohydrates from exposure and degradation by pathogen-sourced acids, ROS, and cell wall degrading enzymes. This water-exclusion mechanism of high S monomer lignin may be supported by an earlier study, where high S/G ratio lignin was reported to be more linear (Kishimoto et al., 2005). However, the exact compositions and subsequent chemical functions of a “more linear” high S/G ratio lignin likely differ in dicots versus monocots due to the differences in hydroxycinnamic acid content found in grass cell walls. Increased incorporation of *p*CA into the lignin polymer appears to reduce lignin recalcitrance, as overexpression of *B. distachyon BdPMT1* in a *BdF5H* overexpression background of poplar (*Populus tremula × Populus alba*) significantly increased terminal integration of free phenolic groups into the transgenic lignin polymer, improving susceptibility to cold alkali pretreatment (Lapierre et al., 2021). Thus, future *in silico* simulations of lignin polymer interactions will likely be capable of testing increasingly complex lignocellulose matrices, enabling investigation of how high S/G ratios impact lignin interactions with cell wall carbohydrates in grasses and dicots, how changes in esterified *p*CA influence diFA crosslinking frequency, or how tricin influences polymerization and lignin association via water exclusion analysis.

#### 3.2.2 PTAL may contribute a significant role in stress lignin synthesis

Biotrophic and necrotrophic resistant cereals have been observed to differentially upregulate lignin biosynthesis genes necessary for stress lignin synthesis, providing genetic support that stress lignin serves a protective function from chemical and enzymatic degradation (**Table 2**). *Triticum monococcum* lines resistant to the biotroph *Blumeria graminis f.* sp. *tritici* (powdery mildew) showed increased expression of *PAL*, *CCoAOMT*, *COMT*, *F5H*, and *CAD* after infection; and increased *B. graminis* susceptibility was observed after both RNAi-knockdown and chemical inhibition of these upregulated genes (Bhuiyan et al., 2009). A later quantitative trait loci analysis of a *F. graminearum-*resistant *T. aestivum* line found upregulation of *PAL, CHS, COMT, CAD*, two *LACs*, and one PRX after pathogen inoculation (Dhokane et al., 2016). In *F. graminearum-* resistant *H. vulgare*, significant upregulation of *PAL, CHS*, and *HCT* in spikelet tissue was observed after infection (Karre et al., 2017). In a subsequent study, similar results from RNAseq of *T. aestivum* seedlings found significant upregulation of *PAL* after *Fusarium pseudograminearum* (Fusarium crown rot) inoculation (Powell et al., 2017). In a recent meta-analysis, all copies of the *PAL* and *C4H* genes in *Z. mays* were consistently upregulated in seven fungal pathogen-resistant *Z. mays* RNAseq datasets (Wang et al., 2022b). In the same study, *4CL1*, *4CL2*, and *4CL4* showed consistent downregulation in all datasets, while *4CL3* was upregulated in two of the datasets. This meta-analysis suggests that *Z. mays* increases hydroxycinnamic acid synthesis in response to fungal pathogen infection, and is in line with recent metabolic phenylpropanoid flux data quantified in *B. distachyon* (Barros et al., 2022).

A common finding among these grass studies, regardless of host or pathogen species, is the upregulation of *PALs* and *PTALs*. Pathogen-induced expression of *PAL* and *PTAL* genes has been associated with broad-spectrum pathogen resistance in *O. sativa* (Zhou et al., 2018; Yadav et al., 2020). Furthermore, analysis of the metabolic consequences of perturbing *PAL* and *PTAL* in *B. distachyon* hinted to the fact that PTAL may contribute to S monolignol synthesis via an uncharacterized pathway (Barros et al., 2022). Specifically, through ^13^C carbon flux analysis of both *BdPAL2i* and grass-specific *BdPTAL1i* in *B. distachyon*, it was observed that ^13^C-labeled tyrosine exhibited a preferential incorporation (up to 50%) into hydroxycinnamic acids CAF, *p*CA, FA, and SA, with significant incorporation into S monomers (Barros et al., 2016). Conversely, ^13^C-phenylalanine showed a significant preference for incorporation into G monomers. The frequency of PAL/PTAL upregulation in multiple grass species in response to multiple pathogens suggest that PTAL may contribute to a broad-spectrum pathogen resistance by triggering an uncharacterized parallel S monolignol synthesis pathway. Functional studies of PAL/PTAL in *S. bicolor* (Godin et al., 2016; Jun et al., 2018; Simpson et al., 2021), *T. aestivum* (Feduraev et al., 2020), and *B. oldhamii* (Hsieh et al., 2021) could be further leveraged to test how PTAL contributes to pathogen resistance in these and other grass species.

#### 3.2.3 Grass monolignol synthesis enzymes may also function as host-susceptibility factors

Several phenylpropanoid enzymes have been found to function as non-receptor-like susceptibility factors, which play a pivotal role in regulating defense signaling and are often targeted by pathogens to increase their virulence (Saur and Huckelhoven, 2021). Recent studies have found multiple lignin biosynthesis enzymes contribute to pathogen defense-related functions beyond their phenylpropanoid metabolic activity, suggesting that they may be host-susceptibility factors. For example, a meta-analysis of the *O. sativa PAL* gene family made four strong arguments to suggest that *PAL*, and possibly *PTAL*, function as non-receptor-like susceptibility genes by contributing a crucial role in broad-spectrum pathogen resistance: 1) *OsPAL* genes are localized in pathogen resistant-associated QTLs; 2) increased *OsPAL* expression is observed in multiple pathogen resistant *O. sativa* lines; 3) OsPAL regulates SA synthesis in response to multiple pathogens in both aerial and root tissue; and 4) resistance mediated by PAL may be durable due to both *PAL* expression and subsequent phenylpropanoid metabolites being downstream of pathogen recognition mechanisms (Yadav et al., 2020). Finally, genetic or chemical suppression of *PAL* in *B. distachyon* (Cass et al., 2015), *O. sativa* (Huang et al., 2010; Tonnessen et al., 2015), *H. vulgare* (Kruger et al., 2002; Prats et al., 2007), and *T. aestivum* (Bhuiyan et al., 2009) results in increased pathogen susceptibility, further supporting the hypothesis that *PAL*, and possibly *PTAL* are host susceptibility factors.

In addition, *CCR*, *HCT*, *CCoAOMT*, and *CAD* may also be considered host-susceptibility factors. In *O. sativa*, CCR1 was found to bind to OsRac1, a plant defense-related effector found to upregulate ROS via NADPH oxidases in a GTPase-dependent manner (Kawasaki et al., 2006). In the same study, *OsCCR1* was upregulated in response to a sphingolipid elicitor, and constitutive overexpression of *OsRac1* led to increased OsCCR1 activity and increased cell wall lignification. In a fascinating series of studies, CCoAOMT and HCT in *Z. mays* were observed to, both independently and as a complex, modulate activation of the R-gene Rp1-D21 during *Puccinia sorghi* (maize common rust) infection (Wang et al., 2015a; Wang and Balint-Kurti, 2016). In addition, overexpression of *CCoAOMT2* in *Z. mays* led to broad-spectrum pathogen resistance (Yang et al., 2017). Recently, *CCoAOMT* in *Z. mays* was found to be co-expressed with *Rp1-D21* at the border of leaf lesions that underwent HR-mediated programmed cell death, and may contribute a role in modulating cell-autonomous HR-mediated programmed cell death (Karre et al., 2021). *Z. mays* FNSI homologs were found to interact with both the HCT and the Rp*-*D21 CCD21 binding domain when transiently expressed in *Nicotiana benthamiana*, suggesting another pathogen defense-related HR attenuation mechanism that is tied to alternative SA synthesis (Zhu et al., 2020). An earlier study of *R. cerealis*-resistant *T. aestivum* revealed that *TaCAD12* was upregulated in response to the pathogen (Rong et al., 2016). Further functional characterization of *TaCAD12* via overexpression increased resistance to *R. cerealis* and increased co-expression of defense genes (*Defensin, PR10, PR17c*, and *Chitinase1*) and other phenylpropanoid genes (*TaCAD1, TaCCR*, and *TaCOMT1*). Taken together, phenylpropanoid genes may be an emerging subtype of non-receptor-like susceptibility factors. Continued research on phenylpropanoid enzyme protein-protein interactions may lead to engineering novel broad-spectrum lignin-based herbivory and parasite resistant cereal and grass lines.

### 3.3 Lignin-based herbivory and parasite resistance in grasses

#### 3.3.1 Constitutive and induced phenolic-based herbivory resistance observed in grass aerial tissues

Grass secondary cell wall compositions contribute a first line of defense against both chewing and invasive herbivore infestation. Unlike pathogenic microorganisms, some herbivores directly and immediately consume the host plant via laceration, chewing, and swallowing of host tissue, and must contend with the immediate composition of consumed lignocellulose, including tissue-specific basal S/G ratios, grass cell wall crosslinking mediated by FA and diFA, and flavonoid-derived phytotoxins (Santiago et al., 2013). Conversely, invasive herbivores physically and enzymatically penetrate plant tissue and induce cell wall modifications via secreted elicitors to feed from host phloem or parasite-induced specialized feeding sites (Waterman et al., 2019; Grover et al., 2022a). Grasses employ two constitutive phenolic-based strategies to resist damage associated with herbivory: toughness, which is the plants pre-digestive anti-nutrition measured via the herbivore’s ability to penetrate or consume the host plant (Lucas et al., 2000; Clissold, 2007), and post-digestive anti-nutrition, which is a measure of a herbivore’s ability to safely and effectively digest the host plant (Clissold, 2007).

Studies into the grass cell wall components that contribute to and enhance pre-digestive herbivory resistance often converge on phenolic compounds that fortify tissue such as hydroxycinnamic acids, lignin, and flavonoids (Hatfield and Marita, 2010; Santiago et al., 2016). For instance, a comparison of cell wall phenolics in *Sesamia nonagrioides* (Mediterranean corn stalk borer) resistant *Z. mays* lines found cell wall-bound *p*CA and S/G ratios in pith tissue to be inversely correlated with stalk penetration (Gesteiro et al., 2021). This study hypothesizes that *Z. mays* balances the degree of diFA-mediated cell wall crosslinking and *p*CA incorporation as cell wall FA and diFA concentrations were positively correlated with plant height, blooming time, and grain yield. These data suggest increased diFA-mediated crosslinking is associated with growth and grain production at the expense of tissue toughness, whereas cell wall-bound *p*CA and increased S/G lignin ratios significantly contribute pre-digestive anti-nutrition herbivory defense at the expense of total plant height and flowering time.

These data also contextualize previous quantifications of *Z. mays* pith versus rind S/G ratios (1.48 and 1.51), cell wall-bound *p*CA (2.1% and 2.7%), and cell wall-bound FA (1.4% and 1.5%) (Barros-Rios et al., 2011), suggesting that rind tissue is on average tougher than pith tissue, and said increased toughness contributes to increased pre-digestive anti-nutritional herbivory defense. Observed *S. nonagrioides* behavior of preferentially infiltrating *Z. mays* via the base of the internode where the intercalary meristem is located and the cells are the least fortified further support that higher cell wall-bound *p*CA and S/G ratios correlate with tissue toughness and improved pre-digestive herbivory resistance (Barros et al., 2011; Barros-Rios et al., 2011). However, *S. nonagrioides-*resistant *Z. mays* lines with increased diFA in pith tissue reduced stem bore depth by 29%, and recovered fed larvae from the high diFA *Z. mays* population were 30-40% lower weight than recovered larvae from contrasting low diFA *Z. mays* population (Barros-Rios et al., 2015), implying that increased diFA-mediated crosslinking is correlated with post-digestive anti-nutritional herbivory defense. In support of these findings, diFA-mediated cell wall crosslinking was negatively correlated with lignocellulose digestibility (Grabber et al., 1998; Grabber, 2005; Hatfield and Marita, 2010).

Grasses are also capable of detecting and reacting to herbivory through inducing phenolic production and cell wall remodeling (Waterman et al., 2019). Chewing herbivores such as *Spodoptera frugiperda* (fall armyworm) produce saliva containing protein and nonprotein-based herbivore-associated molecular patterns (HAMPs) that have been observed to elicit (Turlings et al., 1993; Chuang et al., 2014; Acevedo et al., 2019; Grover et al., 2022b) and suppress (de Lange et al., 2020; Grover et al., 2022b; Warburton et al., 2023) herbivory defense responses in *Z. mays*, *O. sativa*, and *S. bicolor*. A study of the effects of *S. nonagrioides* regurgitant on *Z. mays* leaves showed no significant change in larvae weight or phenolic cell wall composition, suggesting that herbivore-plant interactions are complex and likely require multiple and specific stimuli to elicit HAMP and ETI responses (Santiago et al., 2017). A comparative study of *S. frugiperda* herbivory on both resistant and susceptible *S. bicolor* lines observed significantly decreased soluble hydroxycinnamic acids *p*CA, CAF, FA, and sinapic acid after infestation (Grover et al., 2022b). Furthermore, the resistant *S. bicolor* line showed increased *FNSII* transcription and increased concentrations soluble and cell wall-bound flavonoids naringenin, apigenin, luteolin, and quercetin in leaf tissue, suggesting that *S. frugiperda* ETI response detection may attenuate phenylpropanoid biosynthesis in favor of flavonoid flux to produce defensive phenolic pesticides. Increased traffic and accumulation of vesicle-like bodies containing H_2_O_2_ and phenolics at plasma membranes of cells neighboring *B*. *graminis* infection in *H. vulgare* leaves suggest antimicrobials are actively transported into the apoplast (An et al., 2006). The observed ETI-mediated prioritization of flavonoid synthesis may be coupled with a similar intracellular transport and apoplastic excretion mechanism, as recently seen in glycosylated monolignol transport in *Physalis pubescens* (Shimada et al., 2021), implying grasses may employ a localized phenolic-based defense mechanism at herbivore feed sites (Jeon et al., 2022). Further research is needed to confirm the identity and characteristics of a model for ETI-induced local synthesis and mobilization of phenylpropanoid-derived pesticides at herbivore feeding sites.

#### 3.3.2 Constitutive and induced root lignification may also contribute to endoparasitic nematode resistance

Invasive parasitic herbivores such as endoparasitic nematodes infest grass root systems in a species-dependent manner, and thus may require species-dependent host defense strategies (Okubara et al., 2019). Three types of endoparasitic nematodes that contribute to significant global cereal crop loss are juvenile root knot nematodes (*Meloidogyne* spp.) (Holbein et al., 2016; Rutter et al., 2022), juvenile cyst nematodes (*Heterodera* spp. and *Globodera* spp.) (Holbein et al., 2016), and root lesion nematodes (*Pratylenchus* spp.) (Fosu-Nyarko and Jones, 2016; Okubara et al., 2019). All three categories of plant endoparasitic nematodes possess a protrusible stylet that aids in physically penetrating plant cell walls. Moreover, all three nematode types secrete a mixture of mostly unidentified effectors and cell wall modifying proteins that facilitate their movement within the host plant, help suppress initial host PTI and subsequent ETI defense responses, and help establish feeding sites that are specific to each nematode species (Wieczorek, 2015; Fosu-Nyarko and Jones, 2016; Holbein et al., 2016).

Emerging research is beginning to suggest that parasitic nematode basal resistance is partially credited to both constitutive and induced root lignification (Okubara et al., 2019). For instance, monocot banana (*Musa* spp.) cultivars resistant to root lesion nematode *Radopholus similis* (burrowing nematode) have significantly more cell wall-bound FA in cortical tissue compared to susceptible lines prior to infestation, and significant 1.4-fold increase in root lignin after infestation (Wuyts et al., 2007). In addition, the differential lignification observed extending from the tracheary elements into the pericycle and endodermal cells immediately neighboring *R. similis* lesion sites were attributed to improved host resistance. Genetic evidence to support endodermal lignification as a significant contributor to nematode-resistance was established after observing *A. thaliana* CS mutant *sgn3-3esb1-1* (no CS lignin) to be significantly more susceptible to *Heterodera schachtii* (cyst nematode) infestation (Holbein et al., 2019). The same study also demonstrated that disruption of CS lignification and CS suberization via *esb1-1CDEF1* (no CS lignin, no CS suberin) significantly increased both *H. schachtti* and *Meloidogyne incognita* (root-knot nematode) susceptibility. The Schengen pathway may contribute a significant defense response that increases endodermal cell toughness, thereby limiting the development of root-knot nematode feeding sites (Doblas et al., 2017; Nakayama et al., 2017; Fujita et al., 2020). Furthermore, previously hypothesized PRX-mediated oxidation of S monomers via *p*CA-S radical transfer (Hatfield et al., 2008) may be key to differentially polymerizing stress lignin at and around root-knot nematode endodermal feeding sites.

Genetic, metabolomic, and histochemical evidence indicate a positive correlation between lignification of roots and nematode resistance. B-aminobutyric acid soil treatment applied to *O. sativa* roots induced resistance to *Meloidogyne graminicola* (root knot nematode) infestation and developmental potential by significantly increasing in *OsPAL* and *Callose Synthase* expression and increasing lignin and callose deposition around *M. graminicola* root galls (Ji et al., 2015). In support of these findings, a *M. graminicola*-resistant variety of *O. glaberrima* was reported to have upregulated phenylpropanoid *(PAL, CCoAOMT, CCR, CAD)*, flavone *(CHS, FNSII)*, and phytohormone (JA, SA, and ethylene) synthesis genes (Petitot et al., 2017). The same study found chemical inhibition of *PAL* activity and JA synthesis increased total nematodes and gall number in chemically treated and infested roots (Petitot et al., 2017)*. O. sativa* RILs resistant to *M. graminicola* were observed to rapidly lignify root epidermal cells after infection, including increased lignification surrounding root galls, resulting in significantly reduced gall numbers (Galeng-Lawilao et al., 2019). In addition, a *T. aestivum* line resistant to *Heterodera avenae* (cereal cyst nematode) showed significant upregulation *PAL* after infestation (Kong et al., 2015). Taken together, grasses seem to employ a localized defensive lignification strategy, reminiscent of pathogen-induced papillae formation (**Figure 4**), that mitigates some types of nematode infections. Further research focusing on mutants in the grass Schengen pathway may aid in the development of more resistant cereals to root knot and cereal cyst nematodes, whereas resistance to root lesion nematodes may be best achieved by increasing epidermal toughness in the root.

## 4 Industrial applications of grass lignin

### 4.1 Advances in grass lignin valorization

#### 4.1.1 Engineering grass lignin qualities to improve saccharification efficiency

Lignin inhibits complete hydrolysis of grass feedstock polysaccharides, and disruption of lignin synthesis improves lignocellulose saccharification efficiencies (Godin et al., 2016; Ralph et al., 2019; Ray et al., 2020; Ha et al., 2021; Ning et al., 2021; Wang et al., 2022a). Molecular simulations have demonstrated that lignin directly and competitively inhibits the recognition mechanism of cellulases (Vermaas et al., 2015). Additionally, *in silico* simulations of lignin-carbohydrate interfaces found that disruption of phenylpropanoid synthesis likely results in disruption of cell wall crosslinking and decreased noncovalent association of lignin polymers to cell wall carbohydrates, thereby decreasing probability of cellulase inhibition (Carmona et al., 2015; Vermaas et al., 2019a). A recent study tested biomass pretreatment methods on a near-homogeneous lignin polymer composed almost entirely of caffeyl lignin (C monomers) (Li et al., 2018b). C-lignin was initially found in *Vanilla planifolia* seed coats (Chen et al., 2012a), and was though to arise by the absence of phenylpropanoid O-methyltransferase activity during seed coat lignin biosynthesis (Li et al., 2018b). C-lignin was posited to be an “ideal lignin” because its near homogeneous composition of β-O-4 bonds may depolymerize into a single product by hydrogenolysis if it remained stable during biomass pretreatment conditions. To test this hypothesis, acid pretreatment was applied to *V. planifolia* seed coats, resulting in near total lignin depolymerization with no detectable undesirable condensation reactions; demonstrating that the theoretical chemistry of an ideal lignin is feasible.

To reduce the energy and resource input cost generated by the necessary pretreatment of plant biomass, genetic engineering strategies have been pursued to alter lignin compositions (Vanholme et al., 2012; Mottiar et al., 2016; Pazhany and Henry, 2019; Ralph et al., 2019; Liu et al., 2021b). Reducing lignin S/G ratios has been consistently linked to improved saccharification efficiency. However, the specific reasons for this correlation may vary depending on the pretreatment methods used in different studies (Christensen and Rasmussen, 2019). *COMT* downregulation in grasses is a reliable genetic strategy to lower S/G ratios in *P. virgatum* (Fu et al., 2011a, b), *B. distachyon* (Ho-Yue-Kuang et al., 2016), a *Saccharum* hybrid (Jung and Altpeter, 2016), *T. aestivum* (Ma and Xu, 2008), *Z. mays* (Piquemal et al., 2002; Pichon et al., 2006; Fornale et al., 2017), and *S. bicolor* (Bout and Vermerris, 2003; Oliver et al., 2005; Saluja et al., 2021). Decreasing COMT activity significantly increases saccharification efficiency by increasing concentrations of 5-hydroxyconiferaldehyde and 5-hydroxyconiferyl alcohol incorporation into the mutant lignin polymer (Green et al., 2014; Ralph et al., 2019). Interestingly, 5-hydroxyconiferyl alcohol preferentially undergoes β–*O*–4 coupling, producing surprisingly linear homopolymers that resemble ideal lignin previously observed in C lignin from *V. planifolia* seed coats (Chen et al., 2012a; Li et al., 2018b; del Río et al., 2022). Reducing *COMT* activity also likely disrupts FA and tricin synthesis (Barros et al., 2019a) (**Figure 1**), thus potentially disrupting AX feruloylation and diFA-mediated cell wall crosslinking (Grabber et al., 1998, 2004; de Oliveira et al., 2015). Indirect evidence suggests the availability of these COMT-derived FA pools are positively correlated with grass lignocellulose recalcitrance, as recent perturbation of *BAHD acyltransferases* resulted in disrupted AX feruloylation and improved lignocellulose saccharification in *S. viridis* (de Souza et al., 2018) and *Saccharum* spp (de Souza et al., 2019). Suppression of one or more of these *BAHD acyltransferases* that mediate AX feruloylation in a *comt* mutant background may test if COMT activity is a significant source of cell wall-bound FA, while also providing additional evidence grass preferential phenylpropanoid metabolism into hydroxycinnamic acid synthesis.

Lastly, lignin polymers have been genetically engineered to contain more β–O–4 bonds in the lignin backbone (Mottiar et al., 2016). These “Zip-Lignins” are a novel class of exotic “designer lignins” that are susceptible to mild alkaline pretreatment while minimally perturbing plant growth and fitness (Wilkerson et al., 2014). A cell wall modeling system in *Z. mays* demonstrated successful polymerization of exogenously supplied G-FA into non-lignified cell walls (Grabber et al., 2008). The ester-interunit conjugation of G-FA was found to readily cleave after mild alkaline pretreatment and was positively correlated with improved saccharification of cell wall carbohydrates, independent of pretreatment. A BAHD acyltransferase from *Angelica sinensis* that generates monolignol ferulate conjugates was characterized as a FMT in poplar (*Populus alba* × *grandidentata*) (Wilkerson et al., 2014). In this study, AsFMT conjugated both G and S monomers to FA. Overexpression of *FMT* led to synthesis of G-FA and S-FA that could be transported, polymerized, and function as Zip-Lignins with no negative growth phenotype. ZmCCR1 substrate specificity for feruloyl-CoA, the ligand for FMT, was successfully leveraged in a genetic engineering strategy to improve lignocellulose digestibility in *Z. mays* (Smith et al., 2017a). The *Zmccr1* mutant showed increased feruloyl-CoA pools, resulting in an increased substrate pool for the native ZmFMT to generate more G-FA and S-FA no significant negative growth phenotype. Recently, grass *FMT* orthologs have been characterized in *P. virgatum* and *S. bicolor* (Smith et al., 2022), demonstrating that a similar genetic engineering strategy may be feasible in other bioenergy grasses.

#### 4.1.2 Prioritizing restrained disruption of monolignol synthesis may mitigate undesirable phenotypes

The adage “too much of a good thing is bad” may apply to genetic engineering of phenylpropanoid metabolism. Altering monolignol biosynthesis in both monocots and dicots can result in unpredictable and sometimes undesirable phenotypes such as dwarfism and sterility (Sattler and Funnell-Harris, 2013; Bonawitz and Chapple, 2013, Ha et al., 2021). For example, *comt* null mutants *brm3* in *Z. mays* (Vignols et al., 1995) and *brm12* in *S. bicolor* (Bout and Vermerris, 2003; Sattler et al., 2012) show decreased biomass and grain yield. In contrast, *Z. mays* knockdown *comt* mutants *brm3* (Piquemal et al., 2002; Pichon et al., 2006) and *S. bicolor brm12* (Green et al., 2014) show no significant decrease in biomass compared to wild type. Similarly, *comt* knockdown mutants in *P. virgatum* (Fu et al., 2011a; Baxter et al., 2014), *T. aestivum* (Ma and Xu, 2008), *H. vulgare* (Lee et al., 2021) and *B. distachyon* (Ho-Yue-Kuang et al., 2016) produce near wild-type biomass. In *O. sativa*, *C3*′*H*-knockout mutants were severely dwarfed and sterile whereas RNAi knockdown mutants reached maturity, set seed, and showed improved saccharification (Takeda et al., 2018). In *Z. mays*, *bm2-bm4* double mutants are dwarfed, show constitutive drought symptoms, and do not reach reproductive maturity, whereas *bm1-bm2-bm3* and *bm1-bm3-bm4* did reach maturity and were similar to wild-type plants (Vermerris et al., 2010). On the other hand, overexpression of transcription factors that regulate lignification in *P. virgatum* also led to dwarfing phenotypes (Rao et al., 2019). Taken together, targeting single or even multiple genes to improve saccharification efficiency of lignocellulose should prioritize reducing, rather than completely removing phenylpropanoid enzyme activity from the grass species of interest (Bonawitz and Chapple, 2010, 2013). Application of restrained disruption is can be found in the commercial success following moderate *CCoAOMT* perturbation of reduced lignin dicot *M. sativa “*HarvXtra,” specifically marketed to livestock and dairy industries (Barros et al., 2019b).

### 4.2 Grass lignin as a green source of complex chemical precursors

The structural complexity of lignin polymers (Ralph, 2010; Ralph et al., 2019; del Río et al., 2022) has ignited research into prospecting plant lignin as an environmentally friendly source of industrial chemical precursors (Calvo-Flores and Dobado, 2010; Zakzeski et al., 2010; Banu et al., 2019; Al-Naji et al., 2022). “Lignin first” biorefineries designed to holistically prioritize lignin, cellulose, and hemicellulose valorization follow published methodology with appropriate standards and references to maximize product yields (Renders et al., 2017; Ruiz et al., 2023). Since grass lignin contains relatively high concentrations of *p*CA, FA, diFA, along with feruloylated and coumaroylated cinnamyl alcohol and carbohydrate conjugates, grass lignin may be a potential source of industrial and pharmaceutical chemical precursors (Islam et al., 2021; Bouyahya et al., 2022). A recent study applied reductive catalytic fractionation (RCF) to *P. virgatum*, *Z. mays* stover, and *T. aestivum* straw to identify the determinants of lignin depolymerization (Chen et al., 2023). The study was able to distinguish core monolignols from pendent phenolate esters conjugated to cell wall carbohydrates. Extraction of chemicals from lignin will likely need to be optimized according to a specific species and tissue type, further stressing the importance of complete cell wall phenolic profiling in future lignin research. Justification for these complex analyses is the potential for discovering natural and renewable sources of pharmaceutical precursors such as the catechol products obtained from *V. planifolia* seed coat lignin (Sedo et al., 2013; Li et al., 2018b). Although artificial catechol synthesis has been reported (Wu et al., 2017), sourcing naturally occurring phenolic precursors as well as genetically engineering existing sources to favor synthesis of desirable phenolics may be cheaper, cleaner, and more productive alternatives to artificial synthesis (Grossman and Vermerris, 2019). Faster, cleaner, and cheaper pretreatment methods of grass lignin containing high concentrations of *p*CA were proposed in *Z. mays* via ozone oxidation of *p*CA-S monolignols (Danby et al., 2018). Lastly, it could be argued that grasses are preferential sources of bioenergy and industrial feedstocks due to their rapid biomass accumulation. Grasses that harbor natural or engineered phenolic profiles can be quickly grown to produce on-demand precursors, thus acting as renewable and sustainable sources of bio-based chemicals.

## 5 Conclusions and future directions

This review provides an overview of the recent progress made in understanding the idiosyncrasies of grass monolignol biosynthesis and how grass lignin contributes to abiotic and biotic stress resistance. Differences between dicot and grass monolignol synthesis are reflective of their divergent anatomical characteristics. For instance, grasses appear to preferentially shunt phenylpropanoid flux into hydroxycinnamic acid metabolism, whereas enzymes responsible for the synthesis of phenylpropanoid shikimate intermediates are necessary for dicot lignification. Furthermore, grasses may possess a parallel yet uncharacterized metabolic S monomer synthesis pathway that contribute to synthesis of *p*CA-S. Grasses may also glycosylate monolignols for storage and transport, but where these conjugation reactions occur, and their exact functions have not been characterized. Moreover, tricin appears to be present in all tested monocots to date, yet the functional role of tricin in grass lignin is currently unknown.

However, the laws of chemistry find common ground between monocot and dicot lignification, such as how small nonpolar monolignols translocate into the apoplast is hypothesized to be facilitated by concentration gradient passive diffusion driven by LAC and PRX polymerization activity, and that monolignols combinatorically polymerize via oxidative radical coupling. Parallel mechanisms that drive Casparian strip development in dicots are quickly being reported in grasses, such as the employment of dirigent proteins that direct the deposition of compositionally different lignin at the Casparian strip compared to surrounding endodermal tissue.

Differences in lignification are also correlated with abiotic stress resistance in both dicots and monocots, but grass studies often do not quantify hydroxycinnamic acid or flavonoid compositions, leaving questions of how these overlooked phenolics may contribute to abiotic stress resistance. Furthermore, the signaling pathways that elicit lignification or changes in lignin in response to abiotic stress in grasses are incidentally reported and are often found not reflective of dicot models. More focus on elucidating grass abiotic stress signaling pathways may lead to discovery of novel quantitative trait loci that can be mapped and selectively bred to improve cereal abiotic stress tolerance. Furthermore, determination of the signaling pathways that govern stress lignin synthesis and deposition may lead to discovery of molecular mechanisms that can be leveraged to artificially prime crops to resist pathogen spread in field conditions. These same mechanisms may also be engineered to favor similar defense responses in flavone signaling that may be tied to the poorly understood mechanisms governing grass monolignol glycosylation, storage, and transport.

Lastly, improving resolution of multi-level omics data and increasingly sophisticated lignocellulose saccharification optimization strategies have led to the development of designer lignins such as Zip Lignins, which reportedly have minimal negative consequences on plant fitness. However, lessons learned from past genetic engineering strategies to improve saccharification suggest future endeavors to employ “restrained disruption” to avoid negative growth phenotypes commonly associated with phenylpropanoid null mutants. Although much has been learned thus far regarding lignification in grasses, future research will most certainly leverage emerging technologies to explore the many uncharacterized mechanisms that drive grass cell wall biology.

## Author contributions

LP: Conceptualization, Visualization, Writing – original draft, Writing – review & editing. RP: Writing – review & editing. JB: Writing – review & editing. LB: Supervision, Writing – review & editing. KS: Funding acquisition, Project administration, Resources, Supervision, Writing – review & editing.
